# Klotho mitigates intervertebral disc degeneration by regulating autophagy and energy metabolism

**DOI:** 10.1002/ctm2.70371

**Published:** 2025-06-13

**Authors:** Md Entaz Bahar, Trang Huyen Lai, Jin Seok Hwang, Quang Nhat Ngo, Rizi Firman Maulidi, Jinsung Yang, Wanil Kim, Seung Pil Yun, Dong‐Kun Lee, June‐Ho Byun, Deok Ryong Kim

**Affiliations:** ^1^ Department of Biochemistry and Convergence Medical Sciences and Institute of Medical Science Gyeongsang National University College of Medicine Jinju‐si Republic of Korea; ^2^ Department of Pharmacology and Convergence Medical Sciences and Institute of Medical Science Gyeongsang National University College of Medicine Jinju‐si Republic of Korea; ^3^ Department of Physiology and Convergence Medical Sciences and Institute of Medical Science Gyeongsang National University College of Medicine Jinju‐si Republic of Korea; ^4^ Department of Oral and Maxillofacial Surgery Institute of Medical Science, College of Medicine Gyeongsang National University Hospital Gyeongsang National University Jinju‐si Republic of Korea

1

Dear Editor,

The reduced proliferation and increased senescence of nucleus pulposus cells (NPCs) are associated with the aging process of intervertebral discs (IVDs), consequently contributing to intervertebral disc degeneration (IVDD),^1^, ^2^, ^3^ and also defective expression of the anti‐aging protein Klotho (KL) is linked to various age‐related diseases, including IVDD.^4^, ^5^, ^6^, ^7^ Our findings indicate that reduced KL expression in NPCs is a key factor leading to altered autophagy, mitochondrial dysfunction, and cellular senescence, offering new insights into the molecular mechanisms behind IVD degeneration.

Given the role of KL protein in cellular senescence, its potential function in IVDD has been proposed (Supplementary file 1, Note S1). We examined gene expression of KL and extracellular matrix (ECM) components in two datasets (GSE122429 and GSE186542) obtained from human NPCs and IVD tissues (Supplementary file 2, Method S1).^8^ NPCs differentiated from embryonic or pluripotent stem cells showed an increase of KL and COL2A1 (Figures 1a–d and S1a–f), indicating potential for intervertebral disc repair (Note S2). By contrast, the gene expression analysis of degenerated IVD tissues exhibited a significant decrease of KL expression, underscoring its vital role in maintaining healthy discs (Figures 1e–g and S1g–i; Note S2).^9^ Additionally, in vivo mice studies showed that aged IVDs were correlated with decreased KL and its co‐receptor FGF‐23 (Supplementary file 3, Method S2, Figures 1h and S2a), as well as increased senescent markers (Figures 1i and S2b), highlighting the impact of KL‐associated cellular aging on IVDD (Note S3).

**FIGURE 1:**
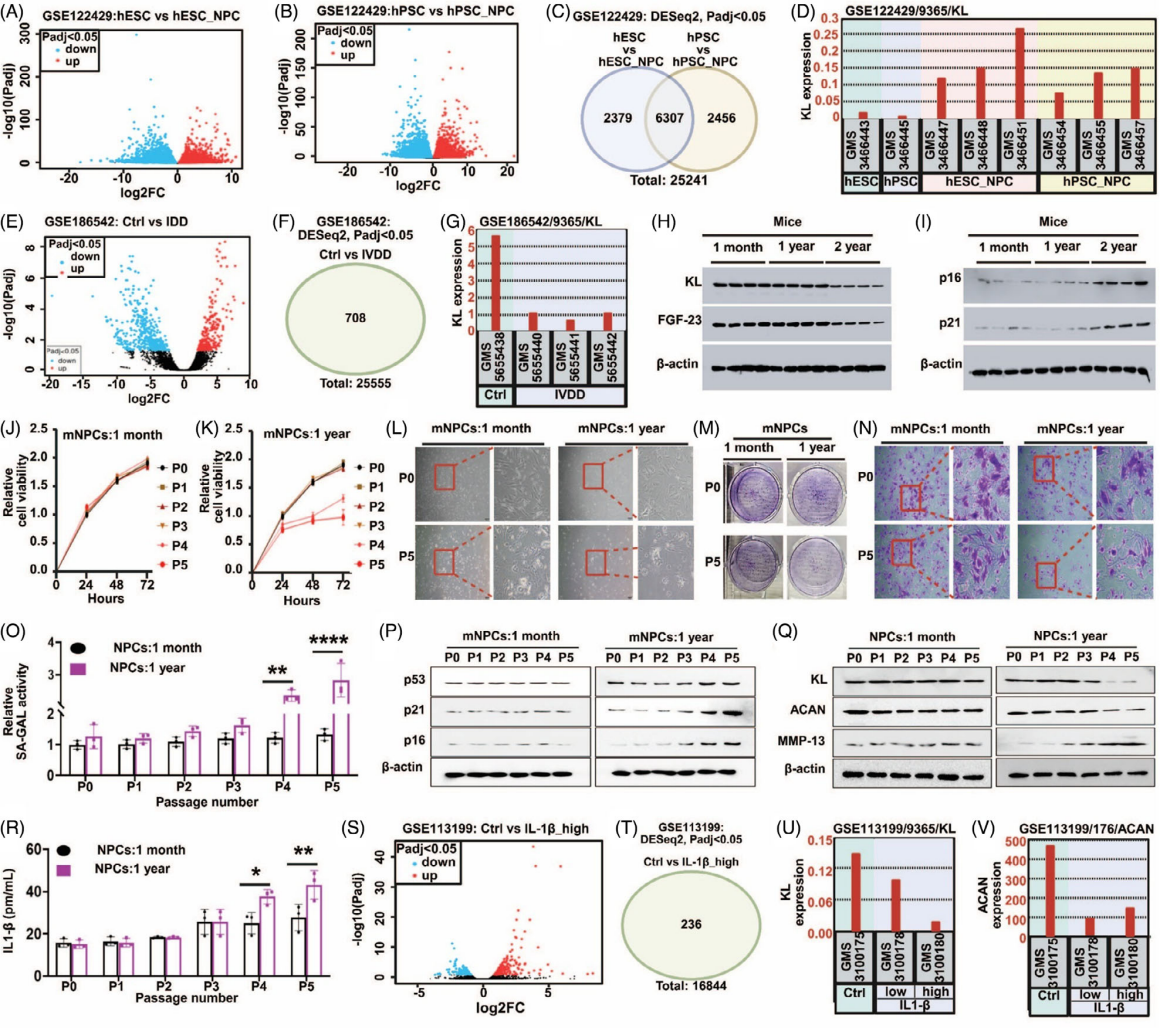
Age‐related changes in gene expression dynamics and therapeutic insights in nucleus pulposus cells (NPCs) and nucleus pulposus tissues. (a, b) Volcano plots illustrating gene expression changes during the differentiation of human embryonic stem cells (hESCs) and pluripotent stem cells (hPSCs) into nucleus pulposus cells (NPCs) using the GSE122429 dataset. (c) Differential expression analysis with DESeq2 identifies 6307 genes involved in the differentiation process, highlighting critical pathways for NPC formation. (d) Klotho (KL) expression is significantly elevated after stem cell therapy, indicating its role in structural repair and enhancement of the disc's metabolic environment. (e) Volcano plot depicting significant gene expression alterations between control and IVDD samples in the GSE186542 dataset. (f) DESeq2 identifies 708 differentially expressed genes from 25 555 analysed. (g) Reduced Klotho expression in degenerative NPCs relative to controls, highlighting its importance in NPC health. (h) Western blot analysis of NP tissues from an in vivo mouse model shows significantly diminished expression of KL and its co‐receptor FGF‐23 in 2‐year‐old mice compared to 1‐month and 1‐year‐old mice. (i) Corresponding increase in senescence markers p16 and p21 in aged mice, indicating enhanced cellular aging and stress. (j, k) Proliferation curves from the CCK‐8 assay showing exponential growth of NPCs from 1‐month‐old mice and reduced growth rates in P4 and P5 cells from 1‐year‐old mice, indicating diminished proliferative capacity with age. (l) The morphological assessment showed regular, spindle‐shaped mNPCs from 1‐month‐old mice across passages, while 1‐year‐old P5 cells displayed more irregular characteristics, suggesting morphological changes with aging. (m) A colony‐forming assay indicated reduced clonogenic potential in P5 cells from 1‐year‐old mice compared to P0, but a decline was not observed in 1‐month‐old mice. (n) Crystal violet assay depicting homogeneous spindle or polygonal shapes in P0 cells from both age groups, with P5 cells from 1‐year‐old mice appearing shorter and less compact yet still forming organised patterns. (o) Enhanced senescence‐associated β‐galactosidase (SA‐β‐GAL) activity in NPCs from 1‐year‐old mice. (p) Elevated levels of senescence markers p53, p21, and p16 in NPCs from 1‐year‐old mice, indicating increased cellular aging and stress. (q) Western blot analyses illustrating reduced expression of Klotho (KL) and aggrecan (ACAN) with increased matrix metalloproteinase‐13 (MMP‐13) levels in NPCs from 1‐year‐old mice, a trend not observed in 1‐month‐old mice. (r) Increased IL1‐β levels in NPCs from 1‐year‐old mice. (s) Volcano plot depicting significant gene expression alterations between control and IL‐1 high‐dose samples in the GSE113199 dataset. (t) DESeq2 identifies 236 genes were differential expression. (u&v) GEO2R analysis from the GSE113199 dataset showing IL1‐β treatment reduces expression of KL and ACAN. Values were represented as mean ± SD, and statistical significance was determined using two‐way ANOVA with Tukey's multiple comparisons in o and r. *p < .05, **p < .01 and ****p < .0001 considered as significantly different.

We further explored the cellular dynamics of mouse NPCs (mNPCs) from the intervertebral lumbar region of older mice (Figure S3a). One‐year‐old mice showed reduced regenerative capacity, with decreased growth rates, irregular morphology, and diminished clonogenic potential at passage 5, while mNPCs from 1‐month‐old mice exhibited strong growth potential and regular morphology from passage 0 to 5 (Figure 1j–n). NPCs derived from older mice also showed the increased activity of senescence‐associated β‐galactosidase (SA‐β‐GAL) and other senescence markers such as p53, p21, and p16, along with higher IL1‐β levels (Figure 1o, p and r). KL and ACAN expression decreased with further passages in older NPCs, and matrix metalloproteinase‐13 (MMP‐13) levels increased, unlike in younger mice's NPCs (Figure 1q). A complementary analysis using the GSE113199 dataset supported these findings, showing that IL1‐β treatment reduced KL, ACAN, and COL2A1 expression (Figures 1s–v and S3b–d). Further details are in Note S4.

Moreover, in vitro cell passaging experiments with human NPCs (hNPCs) demonstrated a reduction in the number of cell population doublings (NCPD) and increased cell population doubling time (CPDT) (Supplementary file 4, Method S3, Figure 2a). Early passage cells (P0 to P5, termed EA‐hNPCs) showed relatively high KL and FGF‐23 protein expression compared to late passage cells (P10 to P13, termed LA‐hNPCs) (Figure 2b). More details are in Note S5.

**FIGURE 2:**
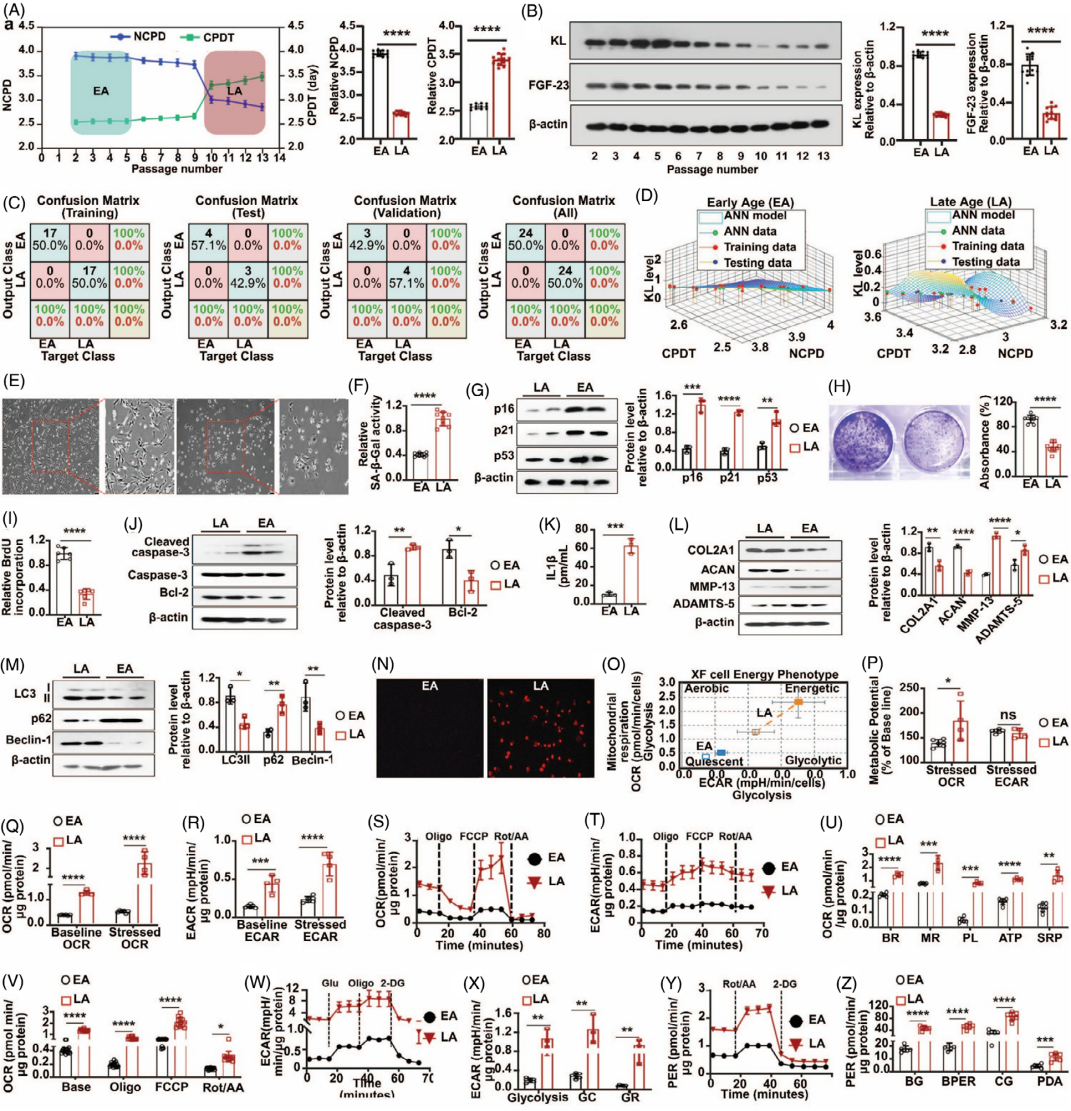
Higher passage hNPCs exhibits lower KL expression, enter towards premature senescence with IVDD phenotypes exhibiting changed bioenergetics profiling (glycolysis and oxidative phosphorylation). (a) Effects of continuous passage on the number of cell population doubling (NCPD) and cell population doubling time (CPDT) of human nucleus pulposus cells (hNPCs) in vitro. The bar graph represents relative NCPD and CPDT in early age (EA) and late age (LA) of hNPCs. (b) The western blotting analysis for measuring protein expression of Klotho (KL) and fibroblast growth factor‐23 (FGF‐23) during continuous passing of hNPCs in vitro (Passage, P2 to P13). The bar graph represents the normalised protein expression of KL and FGF‐23 in EA‐ and LA‐hNPCs. (c) Confusion matrices of training, testing, and validating data classified as predefined labelled classes of EA‐ or LA‐hNPC. (d) The surface graph of the ANN model represents the correlation of KL protein expression, NCPD, and CPDT in EA‐ and LA‐hNPCs. (e) The morphological evaluation of EA‐ and LA‐hNPCs. Scale bar, 50 µm. (f) The senescence‐associated‐β‐galactosidase (SA‐β‐GAL) activity of EA‐ and LA‐hNPCs. (g) The western blotting analysis for measuring senescence proteins, p16, p21, and p53 in EA‐ and LA‐hNPCs. (h) The crystal violet assay shows the clonogenic capability of EA‐ and LA‐hNPCs. (i) The BrdU incorporation capability of EA‐ and LA‐hNPCs. (j) The western blotting analysis for the measurement of apoptosis protein, cleaved caspase‐3 and anti‐apoptotic protein, Bcl‐2 expression in EA‐ and LA‐hNPCs. (k) The measurement of the amount of IL1β in EA‐ and LA‐hNPCs. (l) The western blotting analysis for the measurement of ECM synthesis (COL2A1 and AGCN) and degradation (MMP‐13 and ADAMTS‐5) proteins expression in EA‐ and LA‐hNPCs. (m) The western blotting analysis for measuring autophagy proteins, LC‐3II/I, p61, and Beclin‐1 in EA‐ and LA‐hNPCs. (n) The images represent mitochondrial ROS in EA‐ and LA‐hNPCs. Scale bar, 50 µm. (o) The bioenergetics shifting of hNPCs in EA‐ and LA‐hNPCs. (p) The metabolic potential of hNPCs in EA‐ and LA‐hNPCs. (q) The baseline and stress oxygen consumption rate (OCR) in EA‐ and LA‐hNPCs. (r) The baseline and stress extracellular acidification rate (ECAR) in EA‐ and LA‐hNPCs. (s) The Mito stress test (MST) to measure OCR in EA‐ and LA‐hNPCs. (t) The MST measures ECAR in EA‐ and LA‐hNPCs. (u) Measuring different parameters, including basal respiration (BR) and maximum respiration (MR), proton leak (PL), ATP production, and spare respiratory capacity (SRC) of EA‐ and LA‐hNPCs. (v) The measurement of OCR under basal conditions, upon addition of Oligomycin (Oligo), the rotenone/antimycin (Rote/AA), and FCCP. (w) The Glycolytic stress test (GST) to measure ECAR in EA‐ and LA‐hNPCs. (x) The measurement of different parameters, including glycolysis, glycolytic capacity (GC), and glycolytic reserve (GR) of EA‐ and LA‐hNPCs. (y) The Glycolytic rate assay (GRA) to the measurement of proton efflux rate (PER). (z) The measurement of different parameters, including basal glycolysis (BG), Basal PER (BPER), compensatory glycolysis (CG), and post‐2‐DG acidification (PDG). The normalised protein expression of cleaved caspase‐3 and Bcl‐2 in EA‐ and LA‐hNPCs. Values were represented as mean ± SD and statistical significance was determined using two‐tailed unpaired *t*‐tests in a, b, f, h, I, k, and multiple *t*‐testing with the Benjamini, Krieger, and Yekutieli method in g, j, l, m, p, q, r, u, v, x, z. *p < .05, **p < .01, ***p < .001, and ****p < .0001 considered as significantly different.

We conducted an in‐depth analysis of the neural network involving LA‐hNPCs and healthy EA‐hNPCs, using training data from NCPD, CPDT, and KL protein expression (Supplementary file 5, Method S4). The results confirmed that the model accurately predicted and classified KL expression as stable in EA‐hNPCs but significantly decreased in LA‐hNPCs, correlating with changes in proliferative capacity (Figures 2c and d and S4–S7, Table S1). Further exploration is in Note S6.

The proliferation capability of hNPCs during in vitro culture was assessed by examining cell morphology, BrdU incorporation, and clonogenic ability in EA‐ and LA‐NPCs. Pre‐mature cellular senescence was evaluated by SA‐β‐GAL activity, autophagy flux activity, mitochondrial bioenergetics analysis, and inflammatory markers like IL1β levels with other functional assays (Supplementary file 6, Method S5–S12, Table S2). Indeed, LA‐hNPCs exhibited cell morphological changes, increased senescence and apoptosis, elevated IL1β levels, reduced proliferation rates, and altered ECM expression, resulting in premature senescence and IVDD phenotypes (Figure 2e–l). LA‐hNPCs also showed decreased autophagic activity and increased mitochondrial ROS levels, with a noticeable shift in bioenergetics profiling (Figures 2m–r and S8a and b). Additionally, bioenergetic profiling in LA‐hNPCs represented significant alterations, with an increase in oxidative phosphorylation and glycolytic flux (Figures 2s–z and S8c–e), suggesting a shift in energy metabolism in response to the rising nutritional demands. Details are further in Note S7.

KL knockdown in hNPCs led to diminished cell growth, increased mitochondrial ROSs, and altered respiration (Supplementary file 7, Method S13, Figure 3a–i). Furthermore, reduction of KL expression resulted in decreased autophagy, accelerated senescence, and disrupted ECM balance (Figure 3j–q). These findings highlight the critical role of KL in cellular homeostasis and preventing IVDD. More discussions are in Note S8.

**FIGURE 3:**
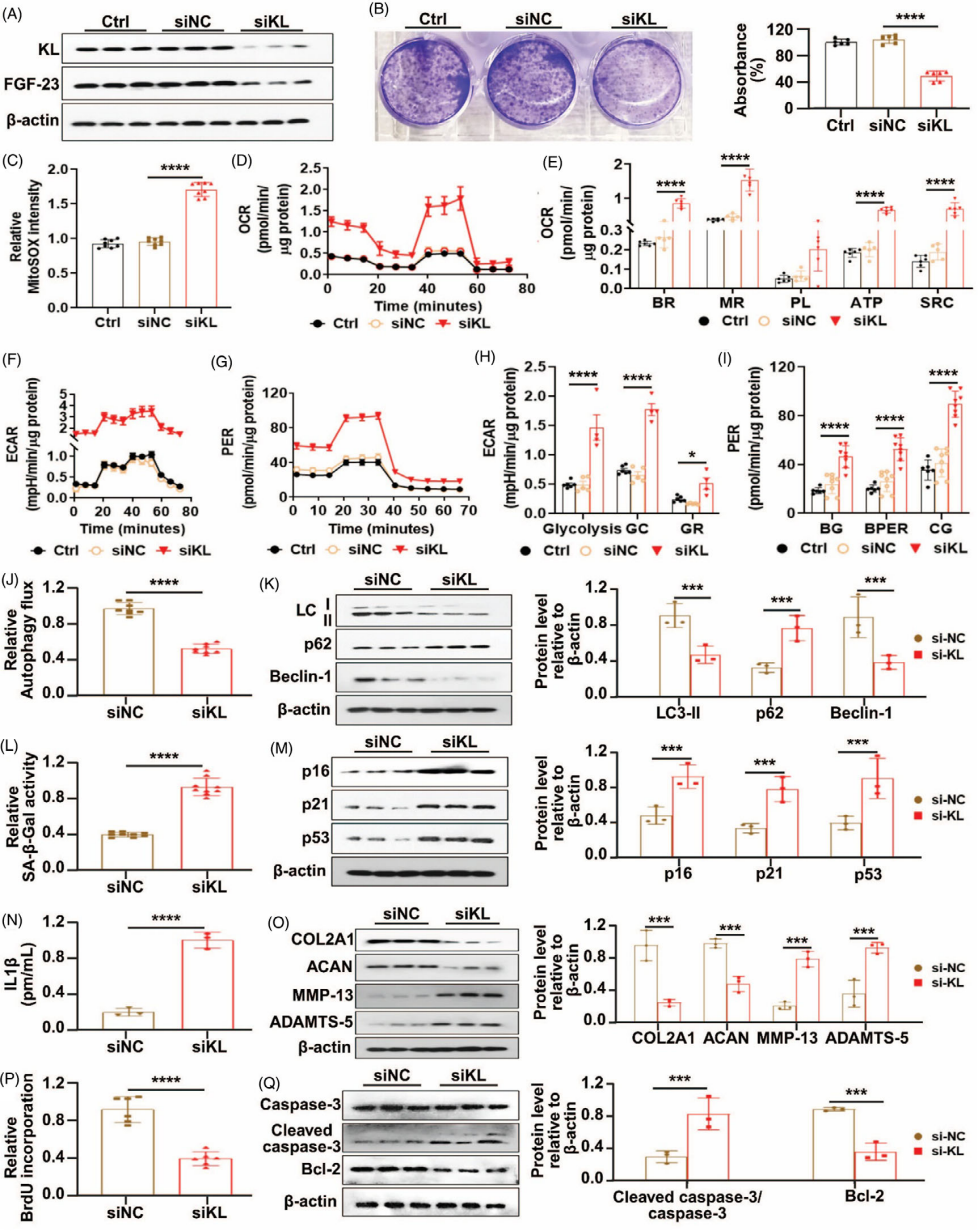
Loss of KL alters cell growth rate and mitochondrial homeostasis with IVDD phenotypes in EA‐hNPCs. (a) The western blotting data for the confirmation of KL knockdown. (b) The measurement of the clonogenic capacity of KL‐deficient hNPCs using crystal violet assay. The bar graph represents the percentage of absorbance in hNPCs at 570 nm. (c) The measurement of mitochondrial ROS in KL‐deficient hNPCs. (d) The oxygen consumption rate (OCR) of KL‐deficient hNPCs. (e) The measurement of different parameters, including basal respiration (BR) and maximum respiration (MR), proton leak (PL), ATP production, and spare respiratory capacity (SRC) of KL‐deficient hNPCs. (f) The extracellular acidification rate (ECAR) of KL‐deficient hNPCs. (g) The measurement of different parameters, including glycolysis, glycolytic capacity (GC), and glycolytic reserve (GR) of KL‐deficient hNPCs. (h) The proton efflux rate (PER) of KL‐deficient hNPCs. (i) The measurement of different parameters, including basal glycolysis (BR), basal PER (BPER), and compensatory glycolysis (CG) of KL‐deficient hNPCs. (j) The measurement of autophagy flux in KL‐deficient hNPCs. (k) The western blotting analysis for measuring autophagy proteins, LC‐3II/I, p61, and Beclin‐1 in KL‐deficient hNPCs. (l) The senescence‐associated‐β‐galactosidase (SA‐β‐GAL) activity of KL‐deficient hNPCs. (m) The western blotting analysis for measuring senescence proteins, p16, p21, and p53 in KL‐deficient hNPCs. (n) The measurement of the amount of IL1β in KL‐deficient hNPCs. (o) The western blotting analysis for measuring ECM synthesis (COL2A1 and AGCN) and degradation (MMP‐13 and ADAMTS‐5) proteins expression in KL‐deficient hNPCs. (p) The BrdU incorporation capability of KL‐deficient hNPCs. (q) The western blotting data for apoptotic protein, cleaved caspase‐3, and anti‐apoptotic protein Bcl‐2). Values were represented as mean ± SD, and statistical significance was determined using ordinary one‐way ANOVA with Tukey's multiple comparisons in b, c, two‐way ANOVA with Tukey's multiple comparisons in e, h, I, k, m, o, q and two‐tailed unpaired *t*‐tests in j, l, n, p. *p < .05, ***p < .001 and ****p < .0001 considered as significantly different.

Additionally, recombinant KL (rKL) treatment exhibited growth potential by enhancing cell proliferation, decreasing oxidative stress, and boosting mitochondrial function in LA‐hNPCs (Supplementary file 8, Method S14, Figures 4a–g and S9a–d). Further results are discussed in Note S9.

**FIGURE 4:**
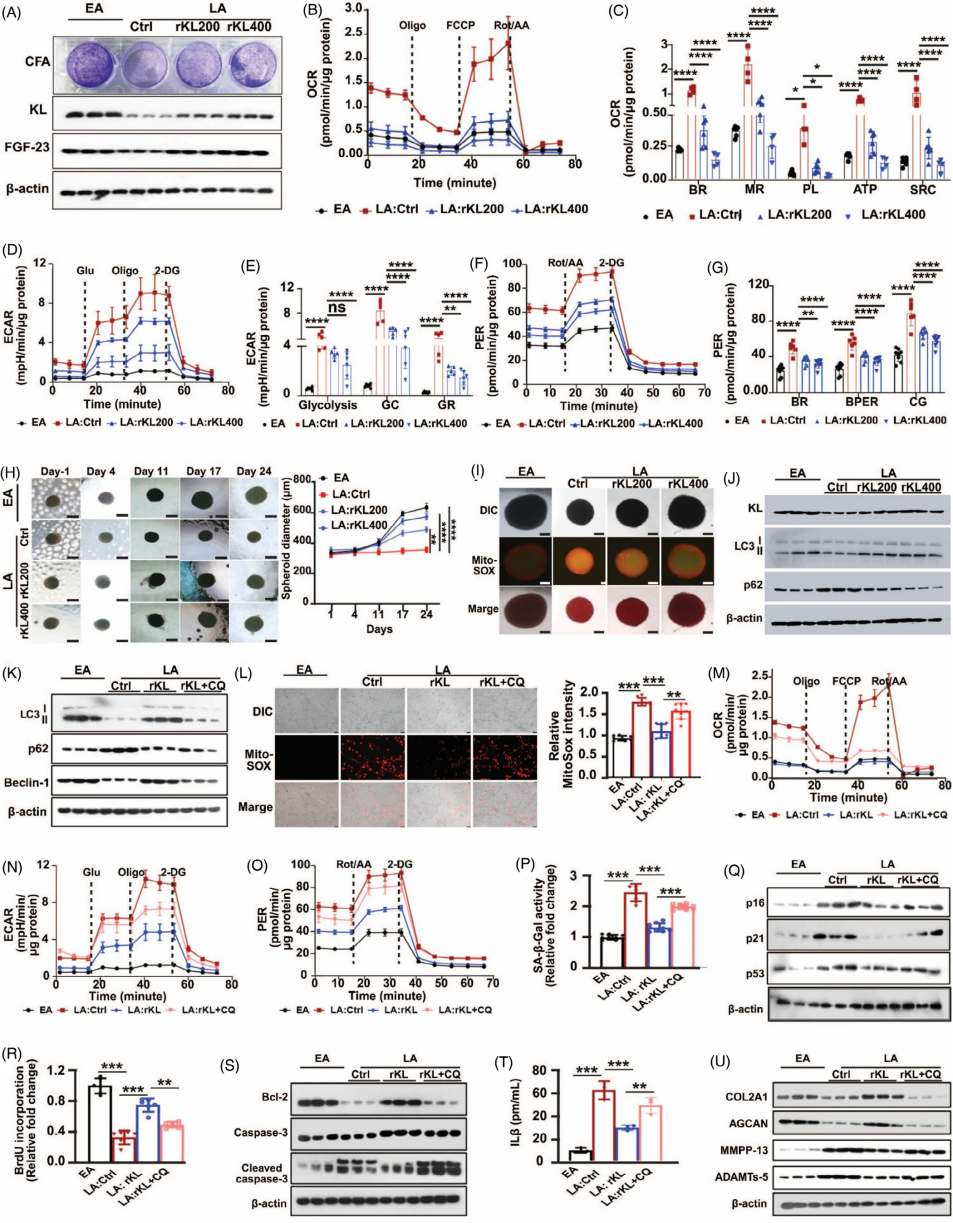
The recombinant KL (rKL)‐induced autophagy reduces premature cell senescence and IVDD phenotypes and restores glycolysis and oxidative phosphorylation homeostasis in LA‐hNPCs. (a) The clonogenic capacity of rKL in LA‐hNPCs and the western blotting data of KL and FGF‐23 for treating rKL in LA‐hNPCs. (b) The oxygen consumption rate (OCR) of LA‐hNPCs after the treatment of rKL. (c) The measurement of different parameters, including basal respiration (BR) and maximum respiration (MR), proton leak (PL), ATP production, and spare respiratory capacity (SRC) of LA‐hNPCs after rKL treatment. (d) The extracellular acidification rate (ECAR) of LA‐hNPCs after the therapy of rKL. (e) The measurement of different parameters, including glycolysis, glycolytic capacity (GC), and glycolytic reserve (GR) of LA‐hNPCs after rKL treatment. (f) The Glycolytic rate assay (GRA) to measure proton efflux rate (PER). (g) The measurement of different parameters, including basal glycolysis (BG), basal PER (BPER), and compensatory glycolysis (CG). (h) Growth patterns of EA and LA spheroids over the experimental timeline. EA spheroids showed a continuous increase in size, as measured on days 1, 4, 11, 17, and 24. (i) Mitosox Red fluorescence intensity analysis. Treatment with rKL significantly reduced Mitosox Red fluorescence intensity, suggesting an antioxidative effect of rKL. (j) Western blot analysis of protein expression in LA spheroids. rKL treatment significantly increased the expression of Klotho (KL) and LC3 II, an autophagy marker, while reducing the expression of p62, associated with autophagic degradation. (k) The western blotting data for autophagy markers, LC‐3I/II, P62, and Beclin‐1 after treating rKL and rKL+CQ in LA of hNPCs. (l) The images represent mitochondrial ROS in LA‐hNPCs after rKL treatment. Scale bar, 50 µm. The bar graph represents the measurement of mitochondrial ROS intensity using MitoSOX dye in LA‐hNPCs after rKL treatment. (m) The oxygen consumption rate (OCR) of LA‐hNPCs after treating rKL and rKL+CQ. (n) The extracellular acidification rate (ECAR) of hNPCs in LA after the therapy of rKL and rKL+CQ. (o) The Glycolytic rate assay to measure the proton efflux rate (PER) of hNPCs in LA after the therapy of rKL and rKL+CQ. (p) The senescence‐associated‐β‐galactosidase (SA‐β‐GAL) activity of LA‐hNPCs when treated with rKL+CQ. (q) The western blotting analysis for measuring senescence proteins, p16, p21, and p53 of LA‐hNPCs when treated with rKL+CQ. (r) The BrdU incorporation ability in of LA‐hNPCs when treated with rKL+CQ. (s) The western blotting data for apoptotic protein, cleaved caspase‐3, and anti‐apoptotic protein Bcl‐2 of LA‐hNPCs when treated with rKL+CQ. (t) The measurement of the amount of IL1b of LA‐hNPCs when treated with rKL+CQ. (u)The western blotting analysis for measuring ECM synthesis (COL2A1 and AGCN) and degradation (MMP‐13 and ADAMTS‐5) proteins expression of LA‐hNPCs when treated with rKL+CQ. Values were represented as mean ± SD and statistical significance was determined using two‐way ANOVA with Tukey's multiple comparisons in c, e, g, h, I, j and ordinary one‐way ANOVA with Tukey's multiple comparisons in l, p, r, t. *p < .05, **p < .01, ***p < .001, and ****p < .0001 considered as significantly different.

In 3D cell culture analyses, EA‐hNPCs spheroids grew steadily, while LA‐hNPCs spheroids lost growth potential (Method S15, Figure 4h). Interestingly, rKL treatment restored growth activity in LA‐hNPCs spheroids. rKL treatment also stimulated antioxidant effects and increased KL expression and autophagy activity (Figures 4i and j and S10a and b), suggesting diverse therapeutic potentials for rKL in controlling spheroid growth dynamics, reducing oxidative stress, and promoting autophagic processes. This is further discussed in Note S10.

Furthermore, co‐treatment with rKL and chloroquine (CQ), an autophagy inhibitor, demonstrated that the protective function associated with KL was impaired due to reduced autophagy, increased mitochondrial ROS, and disrupted mitochondrial homeostasis (Figure 4k–u **)**. Blocking autophagy also reduced rKL‐induced ECM secretion and promoted cellular senescence, suggesting a potential role of rKL in therapeutic effects via autophagy regulation. Further results are in Note S11.

In conclusion, our comprehensive study showed a significant KL role in autophagy, along with metabolic changes in senescent NPCs, including impaired mitochondrial function and increased glycolysis (Supplementary file 9, Note S12, Figure S12). These findings emphasise that targeting KL‐associated pathways could offer therapeutic strategies to delay or prevent IVDD. Notably, rKL therapy emerges as a promising approach for tackling IVDD and potentially alleviating other age‐related diseases.

## AUTHOR CONTRIBUTIONS

M.E.B. conceptualised, wrote the manuscript, and created all original figures. D.S.M. analysed data and edited the manuscript. T.H.L. supported the experiments and edited the manuscript. J.S.H. supported the experiments and edited the manuscript. N.N.Q. and R.F.H. supported the experiments. J.Y. edited the manuscript. W.K. edited the manuscript. D.K.L. edited the manuscript. S.P.Y. edited the manuscript. J.H.B. edited the manuscript, provided financial support. D.R.K. conceptualised, provided financial support, edited all figures, and contributed to writing the manuscript. All authors have read and agreed to the published version of the manuscript.

## CONFLICT OF INTEREST STATEMENT

The authors declare that they have no competing interests

## FUNDING INFORMATION

This study was supported by grants from the Basic Science Research Program through the National Research Foundation of Korea (RS‐2023‐00219399, RS‐2023‐00238051).

## ETHICS STATEMENT

All animal experiments were approved by the Institutional Animal Care and Use Committee (IACUC) of Gyeongsang National University (GNU‐240702‐M0130) and conducted in accordance with the IACUC guidelines of Gyeongsang National University, Republic of Korea.

## Supporting information



Supporting Information

Supporting Information

Supporting Information

Supporting Information

Supporting Information

Supporting Information

Supporting Information

Supporting Information

Supporting Information

## Data Availability

The RNA sequencing datasets analysed during the current study are available in the Gene Expression Omnibus (GEO) repository (GSE11224429, GSE186542, and GSE113199) via the GEO database ((https://www.ncbi.nlm.nih.gov/geo/query/acc.cgi). All data are included in this article and its supplementary information, and they are also available from corresponding author on reasonable request.
