# Supplementary material for: Klotho mitigates intervertebral disc degeneration by regulating autophagy and energy metabolism
Source: Clin Transl Med. 2025 Jun 13;15(6):e70371. doi: 10.1002/ctm2.70371 (PMC12166123; doi:10.1002/ctm2.70371)
Supplement: Supplementary file 1 — Supporting Information [file CTM2-15-e70371-s005.docx]

**Note S1**

Aging is a significant risk factor for numerous chronic diseases ^1^. Among the prevalent age-related ailments, lower back pain emerges as a common affliction, causing considerable distress and functional impairment while imposing a substantial financial burden on society ^2-4^. A major contributor to discogenic lower back pain is intervertebral disc degeneration (IVDD), traditionally associated with diminishing proteoglycan content. This process ultimately leads to endplate sclerosis, osteophyte production, and a decrease in intervertebral height ^5-10^. However, the precise mechanism underlying IVDD remains predominantly unclear, necessitating further exploration for the development of effective treatment approaches.

Conventional treatment methods, such as surgery and conservative therapy, primarily aim at alleviating symptoms rather than fostering regeneration of the damaged disc. As a result, biological strategies that center on restoring the structure and functionality of IVD are considered more promising ^11-14^. The IVD comprises two distinct compartments: the nucleus pulposus (NP), rich in proteoglycans, serving as an internal semi-fluid mass, and the annulus fibrosus (AF), rich in collagen, acting as a laminar fibrous container crucial for maintaining IVD homeostasis ^15-18^. The nucleus pulposus cells play a pivotal role in IVD homeostasis by producing collagen I, collagen II, and proteoglycan, the primary components of the gelatinous structures within NP responsible for ECM balance in the IVD ^19,20^. However, during IVD aging and degeneration, the regular activity of NPCs was hindered, leading to an ECM imbalance that may exacerbate IVDD ^21,22^. Reactivating degenerated NPCs emerges as a promising biological approach for IVDD treatment. Nevertheless, the challenge of producing a sufficient number of cells for repeated clinical applications poses a limitation to in vitro NPCs culture, potentially attributed to replicative senescence during continuous cell passaging. To overcome this impediment in cell growth, we sought to identify the molecular factors contributing to the reduced proliferative ability of NPCs.

Klotho is a transmembrane protein with a single-pass structure, sharing sequence similarities with beta-glucosidase enzymes. It has been associated with the suppression of various aging-related phenotypes, such as shortened lifespan, infertility, arteriosclerosis, skin atrophy, osteoporosis, and emphysema ^23-27^. Subjected to cleavage by proteases, KL yields a cleaved form (cleaved KL), while its transcript is believed to undergo alternative splicing, resulting in a soluble form (secreted KL) ^23,28^. The extracellular polypeptides of Klotho unite to create "soluble -KL," functioning as an endocrine agent with widespread effects ^29^. KL forms complexes with fibroblast growth factor receptors to generate FGF23 co-receptors, facilitating engagement in FGF23-mediated signal transduction and the regulation of phosphate and calcium homeostasis ^30,31^. Displaying diverse effects, KL exhibits antioxidative, anti-senescence, pro-autophagy, anti-apoptosis, anti-fibrosis, pro-stem cell, and anti-insulin properties ^29,32-35^.

The reduced expression of the anti-aging protein KL has been associated with various age-related diseases, including IVDD ^36-39^. Loss of KL protein in mammals can lead to multiple senescence-like symptoms, while its overexpression have been linked to extended lifespan ^23,40-42^. Exogenous KL has been reported to diminish the release of senescence-associated proteins, thereby preserving the survival and phenotypic characteristics of NPCs ^39^.

NCPD = 3.322* [log (N_t_/N_i_)]

CPDT= (t-t_i_)/NCPD

Where N_t_ and N_i_ are the cell numbers at a specific time point t (10 days) and at initial time point N_i_ (0 days), respectively.

The subsequent experiment used cells from the P2-P5 for EA and the P10-P13 for LA.

Several studies have highlighted the interconnectedness of autophagy, cell senescence, and apoptosis in the development of IVDD ^43,44^. Autophagic flux, encompassing the entire dynamic autophagy process, has been implicated in the emergence of IVDD ^45^. Also, some others have demonstrated a correlation between KL expression and alterations in autophagy activity across various disorders ^46-50^. Notably, KL has been found to exert protective properties in regulating autophagy, with higher expression associated with improved abnormal autophagy and lower expression linked to worsened abnormal autophagy ^38,51,52^. However, minimal evidence currently links autophagy with KL in IVDD. Therefore, in this study, we established *in vitro* aging cell models of NPCs to investigate how KL regulates IVDD, with a specific focus on mitochondrial biogenesis, autophagy, apoptosis, cell senescence, and ECM maintenance. Additionally, we assessed the impact of recombinant KL treatment in both preventative and therapeutic contexts, revealing its potential to reduce the onset and progression of IVDD phenotypes and enhance the proliferative capacity during repeated cell passages of NPCs.

**References**

1. Bland JS. Age as a Modifiable Risk Factor for Chronic Disease. *Integr Med (Encinitas)*. Aug 2018;17(4):16-19.

2. de Souza IMB, Sakaguchi TF, Yuan SLK, et al. Prevalence of low back pain in the elderly population: a systematic review. *Clinics (Sao Paulo)*. 2019;74:e789. doi:10.6061/clinics/2019/e789

3. Wong AYL, Karppinen J, Samartzis D. Low back pain in older adults: risk factors, management options and future directions. *Scoliosis Spinal Disord*. 2017;12:14. doi:10.1186/s13013-017-0121-3

4. Katz JN. Lumbar disc disorders and low-back pain: socioeconomic factors and consequences. *J Bone Joint Surg Am*. Apr 2006;88 Suppl 2:21-4. doi:10.2106/JBJS.E.01273

5. Yang S, Zhang F, Ma J, Ding W. Intervertebral disc ageing and degeneration: The antiapoptotic effect of oestrogen. *Ageing Res Rev*. Jan 2020;57:100978. doi:10.1016/j.arr.2019.100978

6. Millecamps M, Stone LS. Delayed onset of persistent discogenic axial and radiating pain after a single-level lumbar intervertebral disc injury in mice. *Pain*. Sep 2018;159(9):1843-1855. doi:10.1097/j.pain.0000000000001284

7. Podichetty VK. The aging spine: the role of inflammatory mediators in intervertebral disc degeneration. *Cell Mol Biol (Noisy-le-grand)*. May 30 2007;53(5):4-18.

8. Wang T, Yang SD, Liu S, Wang H, Liu H, Ding WY. 17beta-Estradiol Inhibites Tumor Necrosis Factor-alpha Induced Apoptosis of Human Nucleus Pulposus Cells via the PI3K/Akt Pathway. *Med Sci Monit*. Nov 12 2016;22:4312-4322. doi:10.12659/MSM.900310

9. Hadjipavlou AG, Tzermiadianos MN, Bogduk N, Zindrick MR. The pathophysiology of disc degeneration: a critical review. *J Bone Joint Surg Br*. Oct 2008;90(10):1261-70. doi:10.1302/0301-620X.90B10.20910

10. Raj PP. Intervertebral disc: anatomy-physiology-pathophysiology-treatment. *Pain Pract*. Jan-Feb 2008;8(1):18-44. doi:10.1111/j.1533-2500.2007.00171.x

11. Ji ML, Jiang H, Zhang XJ, et al. Preclinical development of a microRNA-based therapy for intervertebral disc degeneration. *Nat Commun*. Nov 28 2018;9(1):5051. doi:10.1038/s41467-018-07360-1

12. Hodgkinson T, Shen B, Diwan A, Hoyland JA, Richardson SM. Therapeutic potential of growth differentiation factors in the treatment of degenerative disc diseases. *JOR Spine*. Mar 2019;2(1):e1045. doi:10.1002/jsp2.1045

13. Mern DS, Beierfuss A, Thome C, Hegewald AA. Enhancing human nucleus pulposus cells for biological treatment approaches of degenerative intervertebral disc diseases: a systematic review. *J Tissue Eng Regen Med*. Dec 2014;8(12):925-36. doi:10.1002/term.1583

14. Huang S, Tam V, Cheung KM, et al. Stem cell-based approaches for intervertebral disc regeneration. *Curr Stem Cell Res Ther*. Dec 2011;6(4):317-26. doi:10.2174/157488811797904335

15. Cheng S, Li X, Lin L, et al. Identification of Aberrantly Expressed Genes during Aging in Rat Nucleus Pulposus Cells. *Stem Cells Int*. 2019;2019:2785207. doi:10.1155/2019/2785207

16. Oegema TR, Jr. Biochemistry of the intervertebral disc. *Clin Sports Med*. Jul 1993;12(3):419-39.

17. Colombini A, Lombardi G, Corsi MM, Banfi G. Pathophysiology of the human intervertebral disc. *Int J Biochem Cell Biol*. 2008;40(5):837-42. doi:10.1016/j.biocel.2007.12.011

18. An HS, Thonar EJ, Masuda K. Biological repair of intervertebral disc. *Spine (Phila Pa 1976)*. Aug 1 2003;28(15 Suppl):S86-92. doi:10.1097/01.BRS.0000076904.99434.40

19. Zheng L, Cao Y, Ni S, et al. Ciliary parathyroid hormone signaling activates transforming growth factor-beta to maintain intervertebral disc homeostasis during aging. *Bone Res*. 2018;6:21. doi:10.1038/s41413-018-0022-y

20. Singh K, Masuda K, Thonar EJ, An HS, Cs-Szabo G. Age-related changes in the extracellular matrix of nucleus pulposus and anulus fibrosus of human intervertebral disc. *Spine (Phila Pa 1976)*. Jan 1 2009;34(1):10-6. doi:10.1097/BRS.0b013e31818e5ddd

21. Wang F, Cai F, Shi R, Wang XH, Wu XT. Aging and age related stresses: a senescence mechanism of intervertebral disc degeneration. *Osteoarthritis Cartilage*. Mar 2016;24(3):398-408. doi:10.1016/j.joca.2015.09.019

22. Feng C, Liu H, Yang M, Zhang Y, Huang B, Zhou Y. Disc cell senescence in intervertebral disc degeneration: Causes and molecular pathways. *Cell Cycle*. Jul 2 2016;15(13):1674-84. doi:10.1080/15384101.2016.1152433

23. Kuro-o M, Matsumura Y, Aizawa H, et al. Mutation of the mouse klotho gene leads to a syndrome resembling ageing. *Nature*. Nov 6 1997;390(6655):45-51. doi:10.1038/36285

24. Tohyama O, Imura A, Iwano A, et al. Klotho is a novel beta-glucuronidase capable of hydrolyzing steroid beta-glucuronides. *J Biol Chem*. Mar 12 2004;279(11):9777-84. doi:10.1074/jbc.M312392200

25. Sopjani M, Rinnerthaler M, Kruja J, Dermaku-Sopjani M. Intracellular signaling of the aging suppressor protein Klotho. *Curr Mol Med*. 2015;15(1):27-37. doi:10.2174/1566524015666150114111258

26. Xu Y, Sun Z. Molecular basis of Klotho: from gene to function in aging. *Endocr Rev*. Apr 2015;36(2):174-93. doi:10.1210/er.2013-1079

27. Hu MC, Shi M, Gillings N, et al. Recombinant alpha-Klotho may be prophylactic and therapeutic for acute to chronic kidney disease progression and uremic cardiomyopathy. *Kidney Int*. May 2017;91(5):1104-1114. doi:10.1016/j.kint.2016.10.034

28. Matsumura Y, Aizawa H, Shiraki-Iida T, Nagai R, Kuro-o M, Nabeshima Y. Identification of the human klotho gene and its two transcripts encoding membrane and secreted klotho protein. *Biochem Biophys Res Commun*. Jan 26 1998;242(3):626-30. doi:10.1006/bbrc.1997.8019

29. Hu MC, Shiizaki K, Kuro-o M, Moe OW. Fibroblast growth factor 23 and Klotho: physiology and pathophysiology of an endocrine network of mineral metabolism. *Annu Rev Physiol*. 2013;75:503-33. doi:10.1146/annurev-physiol-030212-183727

30. Kurosu H, Ogawa Y, Miyoshi M, et al. Regulation of fibroblast growth factor-23 signaling by klotho. *J Biol Chem*. Mar 10 2006;281(10):6120-3. doi:10.1074/jbc.C500457200

31. Urakawa I, Yamazaki Y, Shimada T, et al. Klotho converts canonical FGF receptor into a specific receptor for FGF23. *Nature*. Dec 7 2006;444(7120):770-4. doi:10.1038/nature05315

32. Prud'homme GJ, Kurt M, Wang Q. Pathobiology of the Klotho Antiaging Protein and Therapeutic Considerations. *Front Aging*. 2022;3:931331. doi:10.3389/fragi.2022.931331

33. Bian A, Neyra JA, Zhan M, Hu MC. Klotho, stem cells, and aging. *Clin Interv Aging*. 2015;10:1233-43. doi:10.2147/CIA.S84978

34. Strumpf NE, Evans LK. The ethical problems of prolonged physical restraint. *J Gerontol Nurs*. Feb 1991;17(2):27-30. doi:10.3928/0098-9134-19910201-09

35. Ahrens HE, Huettemeister J, Schmidt M, Kaether C, von Maltzahn J. Klotho expression is a prerequisite for proper muscle stem cell function and regeneration of skeletal muscle. *Skelet Muscle*. Jul 4 2018;8(1):20. doi:10.1186/s13395-018-0166-x

36. Lim K, Halim A, Lu TS, Ashworth A, Chong I. Klotho: A Major Shareholder in Vascular Aging Enterprises. *Int J Mol Sci*. Sep 19 2019;20(18)doi:10.3390/ijms20184637

37. Zhou HJ, Zeng CY, Yang TT, Long FY, Kuang X, Du JR. Lentivirus-mediated klotho up-regulation improves aging-related memory deficits and oxidative stress in senescence-accelerated mouse prone-8 mice. *Life Sci*. May 1 2018;200:56-62. doi:10.1016/j.lfs.2018.03.027

38. Zhou H, Pu S, Zhou H, Guo Y. Klotho as Potential Autophagy Regulator and Therapeutic Target. *Front Pharmacol*. 2021;12:755366. doi:10.3389/fphar.2021.755366

39. Yi YY, Chen H, Zhang SB, Xu HW, Fang XY, Wang SJ. Exogenous Klotho ameliorates extracellular matrix degradation and angiogenesis in intervertebral disc degeneration via inhibition of the Rac1/PAK1/MMP-2 signaling axis. *Mech Ageing Dev*. Oct 2022;207:111715. doi:10.1016/j.mad.2022.111715

40. Masuda H, Chikuda H, Suga T, Kawaguchi H, Kuro-o M. Regulation of multiple ageing-like phenotypes by inducible klotho gene expression in klotho mutant mice. *Mech Ageing Dev*. Dec 2005;126(12):1274-83. doi:10.1016/j.mad.2005.07.007

41. Torres PU, Prie D, Molina-Bletry V, Beck L, Silve C, Friedlander G. Klotho: an antiaging protein involved in mineral and vitamin D metabolism. *Kidney Int*. Apr 2007;71(8):730-7. doi:10.1038/sj.ki.5002163

42. Yamashita T, Nifuji A, Furuya K, Nabeshima Y, Noda M. Elongation of the epiphyseal trabecular bone in transgenic mice carrying a klotho gene locus mutation that leads to a syndrome resembling aging. *J Endocrinol*. Oct 1998;159(1):1-8. doi:10.1677/joe.0.1590001

43. Zheng G, Pan Z, Zhan Y, et al. TFEB protects nucleus pulposus cells against apoptosis and senescence via restoring autophagic flux. *Osteoarthritis Cartilage*. Feb 2019;27(2):347-357. doi:10.1016/j.joca.2018.10.011

44. Hu S, Chen L, Al Mamun A, et al. The therapeutic effect of TBK1 in intervertebral disc degeneration via coordinating selective autophagy and autophagic functions. *J Adv Res*. May 2021;30:1-13. doi:10.1016/j.jare.2020.08.011

45. Kang L, Xiang Q, Zhan S, et al. Restoration of Autophagic Flux Rescues Oxidative Damage and Mitochondrial Dysfunction to Protect against Intervertebral Disc Degeneration. *Oxid Med Cell Longev*. 2019;2019:7810320. doi:10.1155/2019/7810320

46. Zeng CY, Yang TT, Zhou HJ, et al. Lentiviral vector-mediated overexpression of Klotho in the brain improves Alzheimer's disease-like pathology and cognitive deficits in mice. *Neurobiol Aging*. Jun 2019;78:18-28. doi:10.1016/j.neurobiolaging.2019.02.003

47. Li P, Shi M, Maique J, et al. Beclin 1/Bcl-2 complex-dependent autophagy activity modulates renal susceptibility to ischemia-reperfusion injury and mediates renoprotection by Klotho. *Am J Physiol Renal Physiol*. Mar 1 2020;318(3):F772-F792. doi:10.1152/ajprenal.00504.2019

48. Lin Y, Sun Z. In vivo pancreatic beta-cell-specific expression of antiaging gene Klotho: a novel approach for preserving beta-cells in type 2 diabetes. *Diabetes*. Apr 2015;64(4):1444-58. doi:10.2337/db14-0632

49. Monick MM, Powers LS, Walters K, et al. Identification of an autophagy defect in smokers' alveolar macrophages. *J Immunol*. Nov 1 2010;185(9):5425-35. doi:10.4049/jimmunol.1001603

50. Shi M, Flores B, Gillings N, et al. alphaKlotho Mitigates Progression of AKI to CKD through Activation of Autophagy. *J Am Soc Nephrol*. Aug 2016;27(8):2331-45. doi:10.1681/ASN.2015060613

51. Manya H, Akasaka-Manya K, Endo T. Klotho protein deficiency and aging. *Geriatr Gerontol Int*. Jul 2010;10 Suppl 1:S80-7. doi:10.1111/j.1447-0594.2010.00596.x

52. Zhao Y, Zeng CY, Li XH, Yang TT, Kuang X, Du JR. Klotho overexpression improves amyloid-beta clearance and cognition in the APP/PS1 mouse model of Alzheimer's disease. *Aging Cell*. Sep 21 2020;19(10):e13239. doi:10.1111/acel.13239
