# Supplementary material for: Klotho mitigates intervertebral disc degeneration by regulating autophagy and energy metabolism
Source: Clin Transl Med. 2025 Jun 13;15(6):e70371. doi: 10.1002/ctm2.70371 (PMC12166123; doi:10.1002/ctm2.70371)
Supplement: Supplementary file 2 — Supporting Information [file CTM2-15-e70371-s003.docx]

**Method S1**

**Data acquisition and analysis**

RNA sequencing (RNAseq) data were obtained from the Gene Expression Omnibus (GEO) database, referencing three specific datasets: GSE11224429, GSE186542, and GSE113199. These datasets were selected based on their relevance to our study objectives. For the dataset GSE11224429, a subset of samples was chosen for analysis: GMS3466443, GMS3466445, GMS3466447, GMS3466448, GMS3466451, GMS3466454, GMS3466455, and GMS3466457. Similarly, for GSE186542, we focused on the samples GMS5655438, GMS5655440, GMS5655441, and GMS5655442. Finally, from GSE113199, the samples GMS3100175, GMS3100178, and GMS3100180 were selected for further analysis. The original datasets were analyzed using the GEO2R tool, a web application provided by the National Center for Biotechnology Information (NCBI) for the analysis of GEO datasets that allows users to compare two or more groups of samples in a GEO Series in order to identify differentially expressed genes. GEO2R utilizes the limma package from Bioconductor to perform its analyses, enabling us to identify significant gene expression changes across the selected samples. The selection criteria for differentially expressed genes included an adjusted p-value threshold to control the false discovery rate, ensuring robust and reliable results. This bioinformatics approach enabled a comprehensive analysis of the RNAseq data, facilitating the identification of key gene expression patterns relevant to our study.

**Note S2**

The first dataset (GSE122429), which has been previously suggested a significant insights into the therapeutic applications of NPCs generated from human embryonic stem cells (hESCs) and pluripotent stem cells (hPSCs)^1^, showed gene expression changes during the differentiation process (**Figure 1a&b**) and identifying 6,307 commonly differentially expressed genes in both cases (**Figure 1c**), highlighting critical pathways for NPC differentiation. Moreover, differentiation from hPSCs and hESCs showed distinct characteristics, with 2,379 and 2,456 differential genes respectively (**Figure 1c**). Particularly, GEO2R analysis showed a significant increase in COL2A1 expression in NPCs from hESCs, suggesting that stem cell therapy could enhance disc matrix by boosting collagen type II synthesis (**Figure S1a**). Klotho expression was notably higher in NPCs from hESCs (**Figure 1d**), indicating a possible role in structural repair and metabolic enhancement in IVD tissues. The gene expression changes were depicted through adjusted p-values (Padj), highlighting genes with significant statistical relevance (Padj < 0.05) (**Figure S1b**) and using a mean difference (MA) plot (**Figure S1c&d**) with color-coding for significant gene expression based on Padj-values. The UMAP map demonstrated unique gene expression profiles between pluripotent stem cells and their NPC descendants (**Figure S1e**), with a box plot confirming consistent expression data across samples (**Figure S1f**). The second dataset (GSE186542), compared degenerative NPCs to controls (**Figure 1e&f**)^2^, showed a significant reduction in Klotho expression in degenerative IVD, underscoring Klotho's role in healthy NPCs (**Figure. 1g**). The MA, UMAP and box plots highlighted gene expression differences between IVDD and controls (**Figure S1g-i**).


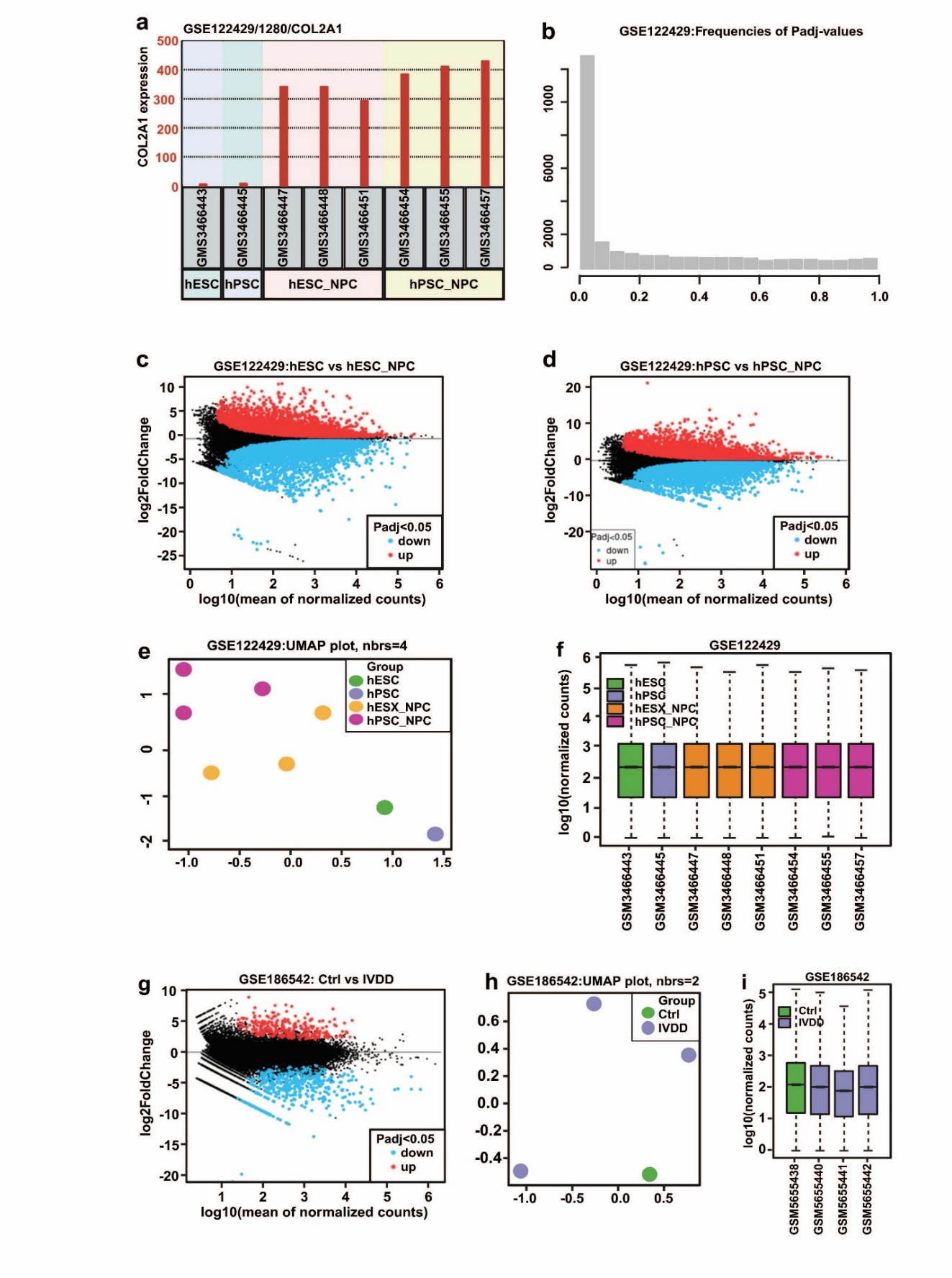
 **FigureS1.** **Gene expression dynamics and therapeutic insights in nucleus pulposus cells (NPCs).** (**a**) Isolation of mouse nucleus pulposus cells (mNPCs) from the lumbar region. (**b**) Distribution of adjusted p-values (Padj), highlighting several genes with substantial statistical significance (Padj < 0.05). (**c, d**) MA plot with color-coding denoting significantly differentially expressed genes according to their Padj values. (**e**) UMAP visualization indicates distinct gene expression profiles between pluripotent stem cells and NPC derivatives. (**f**) Box plot corroborating uniform expression data across samples. (**g**) MA plot with color-coding denoting significantly differentially expressed genes according to their Padj values. (**h**) UMAP plot demonstrates apparent clustering of control and IVDD samples, signifying substantial gene expression disparities. (**i**) Box plot verifying successful data normalization, facilitating impartial comparisons.

**References**

1. Zhang YL, Zhang Z, Chen PK, et al. Directed Differentiation of Notochord-like and Nucleus Pulposus-like Cells Using Human Pluripotent Stem Cells. *Cell Rep*. Feb 25 2020;30(8):2791-+. doi:10.1016/j.celrep.2020.01.100

2. Wu YJ, Li S, Shen JL, Wang ZY, Liu H. Nucleus pulposus related lncRNA and mRNA expression profiles in intervertebral disc degeneration. *Genomics*. Mar 2023;115(2)doi:ARTN 11057010.1016/j.ygeno.2023.110570
