# Supplementary material for: Klotho mitigates intervertebral disc degeneration by regulating autophagy and energy metabolism
Source: Clin Transl Med. 2025 Jun 13;15(6):e70371. doi: 10.1002/ctm2.70371 (PMC12166123; doi:10.1002/ctm2.70371)
Supplement: Supplementary file 3 — Supporting Information [file CTM2-15-e70371-s009.docx]

**Method S2**

**Animals and isolation of mouse nucleus pulposus cells**

Two type of male C57BL/6 mice: one-month-old (20–22 g) and one or two-year-old (30-35 g), were obtained from Hana Biotech (Republic of Korea). The mice were maintained under standard laboratory conditions, a 12-hour light/dark cycle, a temperature of 22°C ± 2°C, and a humidity level of 50% ± 5%. They were provided with a standard laboratory diet and water freely. All animal experiments were approved by the Institutional Animal Care and Use Committee (IACUC) of Gyeongsang National University (GNU-240702-M0130) and conducted in accordance with the IACUC guidelines of Gyeongsang National University, Republic of Korea.

NCPD = 3.322* [log (N_t_/N_i_)]

CPDT= (t-t_i_)/NCPD

Where N_t_ and N_i_ are the cell numbers at a specific time point t (10 days) and at initial time point N_i_ (0 days), respectively.

The subsequent experiment used cells from the P2-P5 for EA and the P10-P13 for LA.

Mouse NP cells (mNPCs) were isolated as previously described^1^. Briefly, the mice were euthanized, and the lumbar spine segments were dissected under sterile conditions using an Olympus SZX16 micro-dissection stereomicroscope. NP-rich IVDs from the lumbar region were isolated while keeping the IVD intact. The dissected IVDs were briefly rinsed in sterile PBS, and the visible gelatinous outpouching NPs were immediately collected in 1.5 mL microcentrifuge tubes. The isolated NP tissues were digested with dispase II (#D4693-1G, Sigma) and collagenase D (#11088866001, Roche) at 37°C for one hour at 150 rpm. After digestion, the mNPCs were filtered through a 40 µM pore-size mesh and centrifuged at 2500 rpm, followed by culturing on collagen-coated 6-well plates (SPL Life Sciences, Republic of Korea) in Dulbecco’s Modified Eagle’s Medium (DMEM; Gibco; Thermo Fisher Scientific, Inc., Waltham, MA, USA) supplemented with 10% fetal bovine serum (FBS, Thermo Fisher Scientific, Waltham, MA) and 1% antibiotic/antimycotic solution (A/A, Sigma-Aldrich) under a 5% CO2 and 5% oxygen atmosphere at 37°C. Primary NP cells were cultured in 6-well plates with different media in duplicates until passage 5 (P5) under consistent culture conditions (37°C, 5% CO2, 95% humidity). The medium was replaced every second day. For sub-cultivation, the cells were detached with trypsin/ethylenediaminetetraacetic acid (Biochrom) on day 6 of passage P0 and every three days for each subsequent passage (P1 to P5). The seeding density was consistently maintained at 5000 cells/cm².

NCPD = 3.322* [log (N_t_/N_i_)]

CPDT= (t-t_i_)/NCPD

Where N_t_ and N_i_ are the cell numbers at a specific time point t (10 days) and at initial time point N_i_ (0 days), respectively.

The subsequent experiment used cells from the P2-P5 for EA and the P10-P13 for LA.

**Note S3**

Klotho and its co-receptor FGF-23 expression in IVD tissues were significantly reduced in two-year-old mice compared to younger mice at 1 month and 1 year (**Figure 1h, Figure S2a**). This decrease corresponded with increased expression of senescence markers p16 and p21 (**Figure 1i, Figure S2b**), indicating enhanced cellular aging and stress in the IVD tissues of older mice.

**Note S4**

To further support the findings in aged mice, we examined cellular dynamics by passaging cells from P0 to P5, which were isolated from one-month-aged young mice or one-year-aged old mice (**Figure S3a**). We did not include two-year-old mice due to the instability and reduced cell survival across multiple passages. Proliferation assessments using growth curves from the CCK-8 assay revealed that NPCs from one-month-old mice underwent an exponential growth phase between 24 and 72 h for P0 to P5 cells (**Figure 1j**). In contrast, P4 and P5 cells from one-year-old mice exhibited lower growth rates during the initial 24 hours and the final 72 h (**Figure 1k**), suggesting a decline in proliferative capacity with age. Morphologically, mNPCs from one-month-old mice retained a regular, homogeneous, and spindle shape from passage 0 (P0) to passage 5 (P5) (**Figure. 1l, left panels**). In contrast, P0 cells from one-year-old mice had a similar morphology to those from younger mice (**Figure 1l, right upper panel**), but by P5, these cells exhibited more irregular, uneven, and detached characteristics (**Figure 1l, right lower panel**), indicating morphological changes with aging. The colony-forming assay showed that P5 cells from one-year-old mice had reduced clonogenic potential compared to P0 cells, a reduction not seen in one-month-old mice (**Figure 1m**), suggesting a decline in regenerative capacity with age. In terms of cell arrangement, the crystal violet assay demonstrated that P0 cells from both age groups formed homogeneous spindle or polygonal shapes, creating vortex patterns **(Figure 1n, upper panel**). However, P5 cells from one-year-old mice appeared shorter and less compact, although they still organized in parallel or spiral formations (**Figure 1n, right lower panel**), whereas P0 cells from one-year-old mice exhibited a pattern similar to their younger counterparts (**Figure 1n, right upper panel**). These findings collectively highlight the impact of aging on NPC morphology, clonogenic potential, and proliferation, emphasizing the challenges in maintaining disc health with advancing age.

In our *in vitro* study, NPCs obtained from one-year-old mice showed a marked increase in senescence-associated β-galactosidase (SA-β-gal) activity (**Figure 1o**), along with higher levels of senescence markers such as p53, p21, and p16, indicating heightened cellular aging and stress (**Figure 1p**). Furthermore, these NPCs exhibited increased levels of IL1-β (**Figure 1r**). As the passage number increased, the expression of KL and aggrecan decreased, whereas matrix metalloproteinase-13 (MMP-13) levels increased, this trend was not observed in NPCs derived from one-month-old mice ((**Figure 1q**). Additionally, a complementary analysis using a public dataset (GSE113199) supports these findings by showng that IL1-β treatment reduced the expression of KL and ACAN (**Figure 1s-v**) and further decreased COL2A1 expression (**Figure S3b**). The reduction of these structural elements under inflammatory conditions suggests the age-related decline consistent with our experiments. The spatial differentiation observed in the UMAP map highlights distinct gene expression profiles between control and IL1-β treated samples **(Figure S3c**), and the box plot confirms consistent expression data across samples (**Figure S3d**). Consequently, the concurrent reduction in KL and matrix components such as ACAN and COL2A1 indicates a shift towards a degenerative state in IVD tissue. This suggests that maintaining KL and matrix constituents could be a potential strategy to mitigate disc degeneration, especially in older populations.


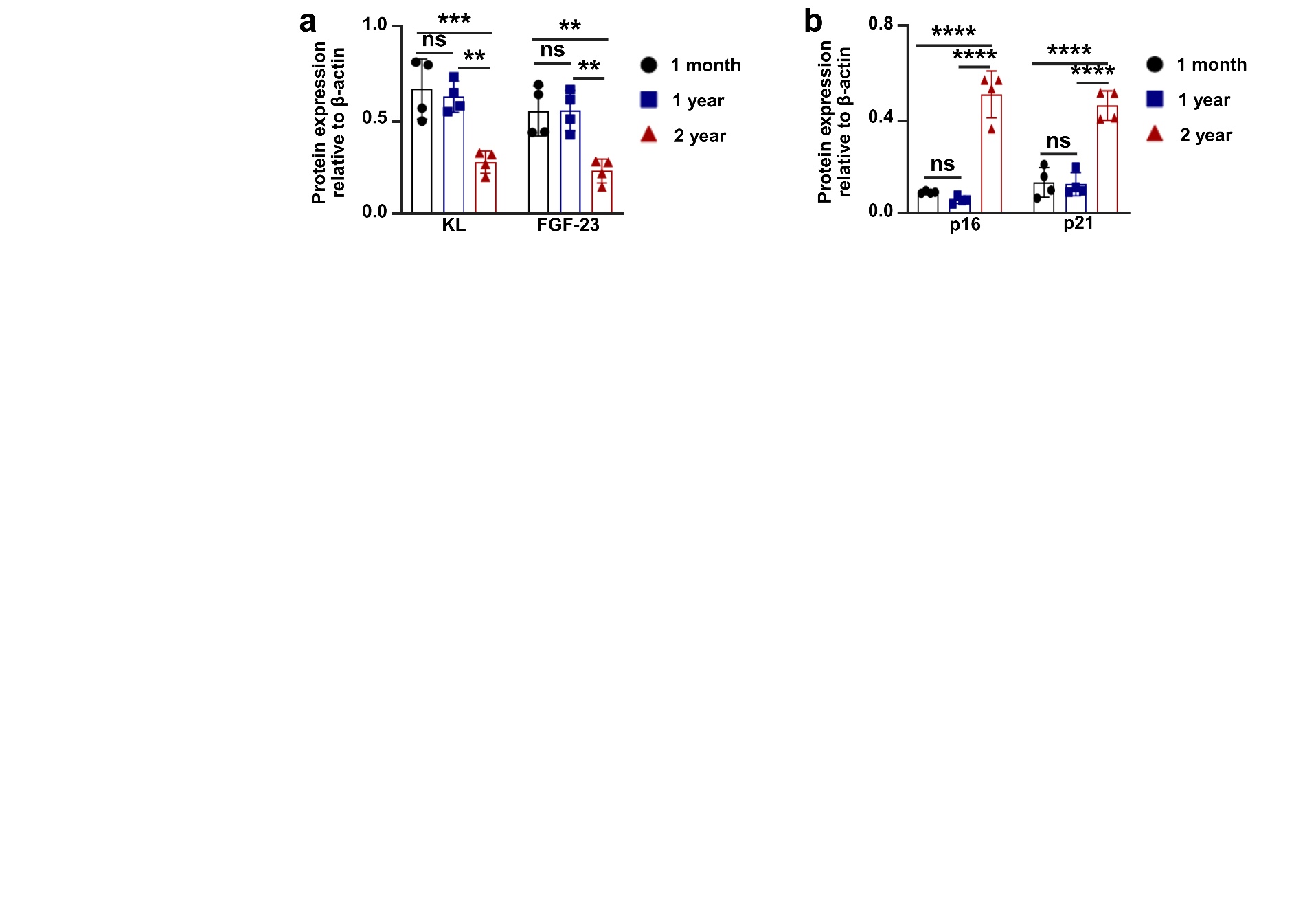
**Figure S2. Age-related changes in cellular dynamics in mouse nucleus pulposus cells (mNPCs).** (**a&b**) Western blot analysis of NP tissues from an in-vivo mouse model shows significantly diminished expression of KL and its co-receptor FGF-23 in two-year-old mice compared to 1-month and 1-year-old mice. Values were represented as mean ± SD, and statistical significance was determined using two-way ANOVA with Tukey’s multiple comparisons in **a** & **b**. ** p < 0.01, *** p < 0.001, and **** p < 0.0001 considered as significantly different. ns considered as not significantly different.


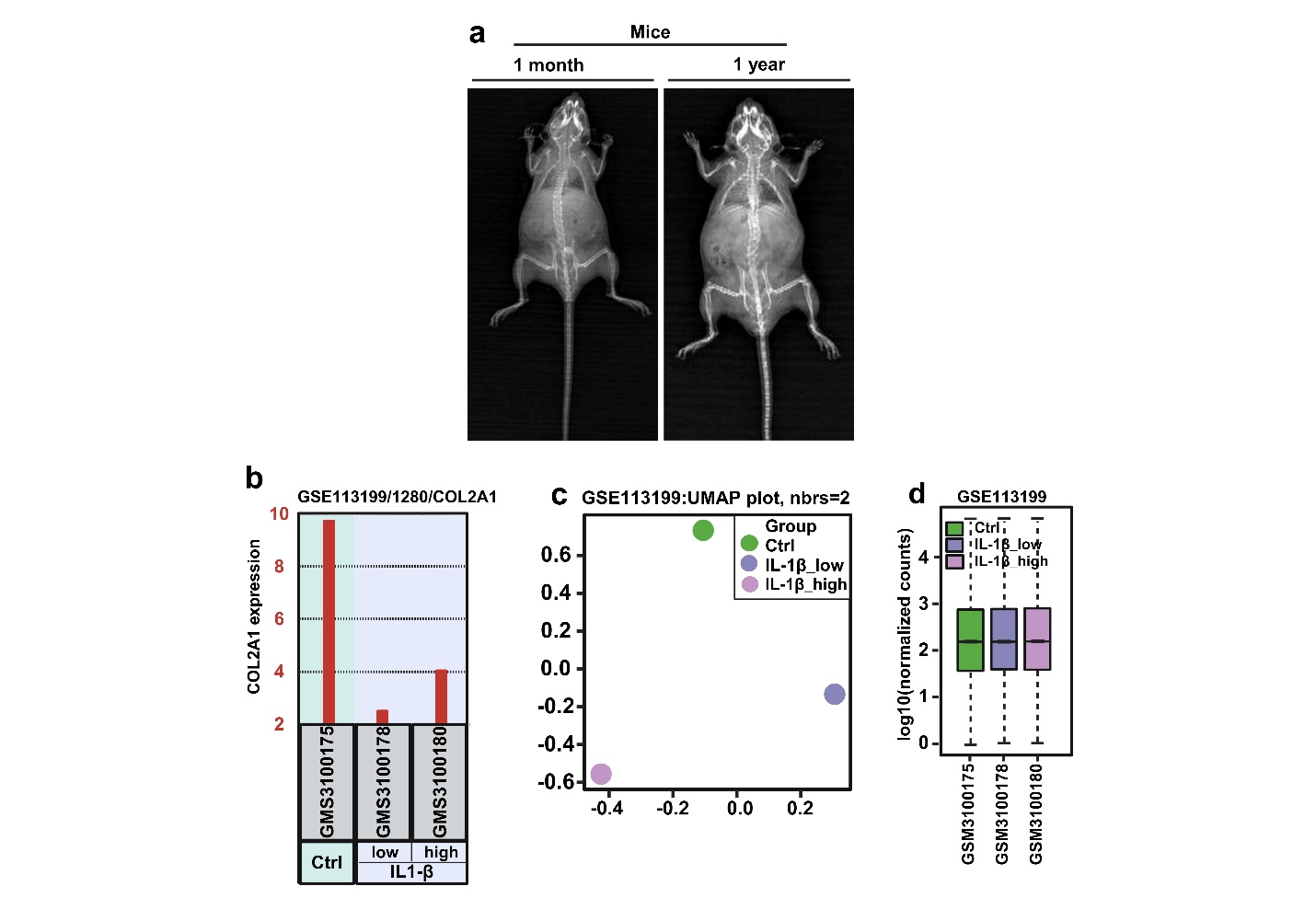
 **Figure S3. Age-related changes in cellular dynamics in mouse nucleus pulposus cells (mNPCs).** (**a**) Isolation of mNPCs from the lumbar region, used for in vitro aging models with cells from 1-month and 1-year-old mice. (**b**) GEO2R analysis from the GSE113199 dataset shows IL1-β treatment reduces COL2A1 expression. (**c**) UMAP plot demonstrates apparent clustering of control, and IL1-β treated samples, signifying substantial gene expression disparities. (d) Box plot verifying successful data normalization, facilitating impartial comparisons.

**References**

1. Bratsman A, Couasnay G, Elefteriou F. A step-by-step protocol for isolation of murine nucleus pulposus cells. *JOR Spine*. Dec 2019;2(4):e1073. doi:10.1002/jsp2.1073
