# Supplementary material for: Klotho mitigates intervertebral disc degeneration by regulating autophagy and energy metabolism
Source: Clin Transl Med. 2025 Jun 13;15(6):e70371. doi: 10.1002/ctm2.70371 (PMC12166123; doi:10.1002/ctm2.70371)
Supplement: Supplementary file 4 — Supporting Information [file CTM2-15-e70371-s002.docx]

**Method S3**

**Determination of numbers of cell population doubling (NCPD) and cell population doubling time (CPDT) of human nucleus pulposus cells (hNPCs)**

Human nucleus pulposus cells (hNPCs) were purchased from ScienCell Research Laboratories, Inc. (#4800, ScienCell, Carlsbad, CA, USA). Cells were cultured in a poly-L-lysine-coated culture plate with Nucleus Pulposus Cell Medium (NPCM, #4801, ScienCell, Carlsbad, CA, USA) supplemented with 2% fetal bovine serum (FBS; #0010, ScienCell, Carlsbad, CA, USA), 1% penicillin/streptomycin (P/S; #0503, ScienCell, Carlsbad, CA, USA), and 1 % nucleus pulposus cell growth supplement (NPCGS; #4852, ScienCell, Carlsbad, CA, USA). To evaluate the phenotypic and morphological changes of *in vitro* aging, hNPCs were seeded on the surface of tissue culture plastics at a constant density of 2500 live cells per cm^2^ and were incubated with NPCM in a humidified incubator (37 °C, 5% CO_2_). The cultural medium was completely replaced with the fresh at 24 hours after cell seeding and every 3 days thereafter. The monolayer NPCs were detached by treatment with 0.025% trypsin at 37 °C for 4 min, and collected cells were passaged at the aforementioned initial cell seeding density every 10 days. Phase-contrast images were captured at each passage to record cell morphology.

NCPD = 3.322* [log (N_t_/N_i_)]

CPDT= (t-t_i_)/NCPD

Where N_t_ and N_i_ are the cell numbers at a specific time point t (10 days) and at initial time point N_i_ (0 days), respectively.

The subsequent experiment used cells from the P2-P5 for EA and the P10-P13 for LA.

The *in vitro* proliferative capability of hNPCs at the end of each passage were determined by Trypan blue exclusion method ^1^. The numbers of cell population doubling (NCPD) ^2^ and cell population doubling time (CPDT) were then calculated based on the following equations (**Eq.**$\boldsymbol{(i}$ **&** $\boldsymbol{ii}$**)**) ^3^:

$$\boldsymbol{NCPD=}\boldsymbol{3.322}^{\boldsymbol{*}}\left( \boldsymbol{logNt-logNi} \right)\boldsymbol{--------------------------}\left( \boldsymbol{i} \right)$$

$$\boldsymbol{CPDT=(t-ti)/NCPD------------------------------ (ii)}$$

Where $\boldsymbol{Nt}$and $\boldsymbol{Ni}$ are the cell numbers at a specific time point $\boldsymbol{t}$ (10 days) and at initial time point $\boldsymbol{ti}$ (0 days), respectively.

EA-hNPCs (passage #2 to 5) and LA-hNPCs (passage #10 to 13) were used for the subsequent experiments.

**Note S5**

In this study, human nucleus pulposus cells at NCPD (number of cell population doubling) around 3.91 and CPDT (cell population-doubling time) around 2.56 h in passage 1 (P1) were utilized. Continuous *in vitro* cell passaging revealed a discernible trend of decreasing NCPD and CPDT as shown in **Figure 2a**. A noteworthy contrast emerged between early passages (P 2 to P5, referred to as EA) and late passages (P10 to P13, denoted as LA) of hNPCs. Late passages exhibited a substantial decrease in NCPD by approximately 23 to 25 %, coupled with an increase in CPDT by around 30 to 35 % (p<0.0001, **Figure 2a**).

To pinpoint the specific molecular mechanisms responsible for the diminished proliferative capacity of hNPCs during *in vitro* cell culture, we assessed the expression of the anti-aging protein KL and its co-receptor FGF-23 across repeated cell passages **(**p<0.0001, **Figure 2b**). The protein expression levels of KL and FGF-23 were found to be downregulated in LA-hNPCs compared to EA-hNPCs (**Figure 2b**).

**References**

1. Strober W. Trypan Blue Exclusion Test of Cell Viability. *Curr Protoc Immunol*. Nov 2 2015;111:A3 B 1-A3 B 3. doi:10.1002/0471142735.ima03bs111

2. Stolzing A, Jones E, McGonagle D, Scutt A. Age-related changes in human bone marrow-derived mesenchymal stem cells: consequences for cell therapies. *Mech Ageing Dev*. Mar 2008;129(3):163-73. doi:10.1016/j.mad.2007.12.002

3. Yang YK, Ogando CR, Wang See C, Chang TY, Barabino GA. Changes in phenotype and differentiation potential of human mesenchymal stem cells aging in vitro. *Stem Cell Res Ther*. May 11 2018;9(1):131. doi:10.1186/s13287-018-0876-3
