# Supplementary material for: Klotho mitigates intervertebral disc degeneration by regulating autophagy and energy metabolism
Source: Clin Transl Med. 2025 Jun 13;15(6):e70371. doi: 10.1002/ctm2.70371 (PMC12166123; doi:10.1002/ctm2.70371)
Supplement: Supplementary file 5 — Supporting Information [file CTM2-15-e70371-s004.docx]

**Method S4**

**Artificial neural network (ANN) modeling approach**

A backpropagation neural network model was constructed by the neural fitting tool (*nftool*) using MATLAB 2019b software. This model was trained with *in vitro* hNPCs propagation parameters as input and the measured KL protein expression level from western blotting as output. The input variables consisted of NCPD and CPDT, while the output variable was the KL protein level. **Sixty** data points from *in vitro* experimental analyses were employed for building the BP neural network model, with the remaining **twelve** data points were used for model verification. In the training, validation, and testing phases of the neural networks, a MATLAB script randomly allocated **forty-two** (70%) of the data for the training subset, **nine** (15%) for the validation subset, and another **nine** (15%) for the test subset (**Figure S4**). Total numbers of neurons and hidden layers were used to determine the overall structure of a BP neural network ^1^. The determination of the number of neurons in the hidden layer was achieved using the empirical formula presented as **Eq. (**$\boldsymbol{iii}$**)**.

$\boldsymbol{h=}\sqrt{\boldsymbol{m+n}}\boldsymbol{+a}\boldsymbol{----------------------------------(iii)}$

The equation involves constants: the number of nodes in the input layer, $\boldsymbol{m}$ ($\boldsymbol{m}$ **= 2**), the number of nodes in the output layer $\boldsymbol{n (n=1)}$, and a (**1 ≤** $\boldsymbol{a}$ **≤ 10**). To determine the number of nodes in the hidden layer, a hit-and-trial approach was adopted, selecting an integer between **two** and **eleven** based on **Eq. (iii).** Bayesian Regularization (BR) and Levenberg-Marquardt (LM) algorithm guided this iterative process. After multiple trials, it was determined that **nine** neurons in the hidden layer, coupled with the LM algorithm, exhibited high accuracy with minimum error. Consequently, a **2-9-1** neural network model was established (**Figure S5a**), wherein **one** neuron in the output layer represented the model's active components, and **two** neurons in the input layer represented the **two** input variables.

To evaluate the overall model’s performance and predictive capacity, metrics such as mean square error (MSE) and root mean square error (RMSE), and mean absolute error (MAE) were employed, as outlined in of **Eq. (**$\boldsymbol{iv}$ ***-*** $\boldsymbol{vi}$**)**.

$\boldsymbol{MSE=}\frac{\boldsymbol{1}}{\boldsymbol{n}}\sum_{\boldsymbol{i=1}}^{\boldsymbol{n}} \boldsymbol{(Xi-Yi)}^{\boldsymbol{2}}\boldsymbol{------------------------------(iv)}$

$$\boldsymbol{RMSE=}\sqrt{\frac{\sum_{\boldsymbol{i=1}}^{\boldsymbol{n}} \left( \boldsymbol{Xi-Yi} \right)^{\boldsymbol{2}}}{\boldsymbol{n}}}\boldsymbol{- ---------------------------(v)}$$

$$\boldsymbol{MAE=}\frac{\sum_{\boldsymbol{i=1}}^{\boldsymbol{n}} \boldsymbol{(Xi-Yi)}^{\boldsymbol{2}}}{\boldsymbol{n}}\boldsymbol{------------------------------(vi)}$$

Where$\boldsymbol{Xi}, \boldsymbol{Yi,}\mathbf{and}\boldsymbol{n}$ in the equation represent the experimental value of the evaluation model, the corresponding predicted data, and is the number of experimental data, respectively.

Furthermore, we employed MATLAB 2019b App, neural network pattern recognition (*nprtool*), which utilizes a two-layer feedforward network for developing a predictive classification model. The model involves one layer for hidden layer activation functions and another for output layer activation functions. In the model, input data (NCPD, CPDT, and KL protein expression level) is categorized into predefined labeled classes to predict the early age (EA) or late age (LA) of hNPCs. The output comprises two classes (EA-hNPC and LA-hNPC), facilitating the classification of different passages of hNPCs. The MATLAB script of *nprtool* partitioned the dataset is into three components: the training dataset, the validation dataset, and the test dataset, with a ratio of 70%, 15%, and 15%, respectively. The *nprtool* employs the Scaled Conjugate Gradient (SCG) algorithm for training ^2^. Optimal results were achieved with **three** neurons in the first layer (input layer), **ten** neurons in the second layer (hidden layer), and **one** neuron in the last layer (output layer). The last layer in the ANN receives input from the hidden layer, undergoes transformations, and outputs a binary result (zero = EA or one = LA). Lower cross entropy values correspond to higher classification accuracy, with zero cross entropy indicating no error. The variation in the gradient coefficient concerning the number of epochs and the minimum value of the gradient coefficient promises enhanced training and testing of the networks.

**NoteS6**

To distinguish between LA-hNPCs and their healthy EA-hNPCs, we employed various neural networks, training them on NCPD, CPDT, and KL protein expression data. Utilizing the MATLAB ANN tools, a backpropagation (BP model) was implemented to predict the data. We evaluated different network structures and optimized configuration, ultimately finding that the 2-9-2 structure effectively predicted both training and testing data (**Figure S5a**). For the training dataset, the MSE, RMSE, and MAE of the entire ANN model were **0.0099, 0.0995,** and **0.0791**, respectively. Corresponding values for the testing dataset were **0.0085, 0923,** and **0.0720** (**Table S1**). The implementation of the MATLAB algorithm using feedforward neural network (FNN) was deemed well-suited for analyzing the aging stages (EA or LA) in hNPCs (**Table S1**). Surface plots illustrated that KL expression remains stable in EA-hNPCs but rapidly decreases in LA-hNPCs, positively correlating with NCPD and negatively correlating with CPDT (**Figure 2d**). The MSE graph for training and testing reached **0.0059815** in 39 epochs **(Figure S5b)**. At epoch 45, the gradient coefficient and Mu value reached **0.010134** and **0.001**, respectively **(Figure S5c)**. A lower gradient value ensures the effective training and testing of the network, with Mu serving as a stop criterion for overtraining. MATLAB automatically ceased training after six consecutive validation failures, as numerous validation fails indicate overtraining. This decision was made based on the iterations where validation MSE increased to zero **(Figure S5c)**. The error histogram displayed minimal discrepancies between target values and the predicted values after training a feedforward neural network, distributed within a narrow range around zero **(Figure S5d)**. We are convinced that our implementation of MATLAB scripts using FNN is well-suited to match the requirements of the prediction of the cell aging stage of hNPCs (**Figure S6**).

In the classification model, the cross-entropy attained a value of **3.603x10^-7^** by the 14th iteration, indicating a notably high classification accuracy **(Figure S7a&b)**. The gradient value exhibited a decreasing trend with an increase in the number of epochs, reaching a final value of the gradient coefficient at the 14th epoch, which was approximately near zero (**8.608x10^-7^**) **(Figure S7c)**. MATLAB initiated an automatic halt to training after 0 validation fails in a row, as a substantial number of validation fails signify overtraining. These iterations occurred when the validation mean square error (MSE) escalated to zero **(Figure S7c)**. The error histogram of the trained neural network illustrated minimal data fitting errors, distributed within a narrow range around zero for the training, validation, and testing segments **(Figure S7d)**. Separately presented were the confusion matrices for the training, validation, and test data **(Figure 2c)**. Simultaneously, the last confusion matrix provided the overall accuracy result of that particular deep neural network (**Figure 2c**).

**Table S1** The performance analysis of the ANN model

|  |  | ***Training*** | | | ***Testing*** | | |
| --- | --- | --- | --- | --- | --- | --- | --- |
|  |  | ***MSE*** | ***RMSE*** | ***MAE*** | ***MSE*** | ***RMSE*** | ***MAE*** |
| ***Overall model*** |  | *0.0099* | *0.0995* | *0.0791* | *0.0085* | *0.0923* | *0.072* |
| ***EA*** |  | *0.0083* | *0.0911* | *0.0784* | *0.0062* | *0.0789* | *0.0692* |
| ***LA*** |  | *0.0034* | *0.0584* | *0.0446* | *0.0034* | *0.0581* | *0.0560* |

***EA: Early age; LA: Late age; MSE: Mean standards error; RMSE: Root mean square error; MAE: Mean absolute error***


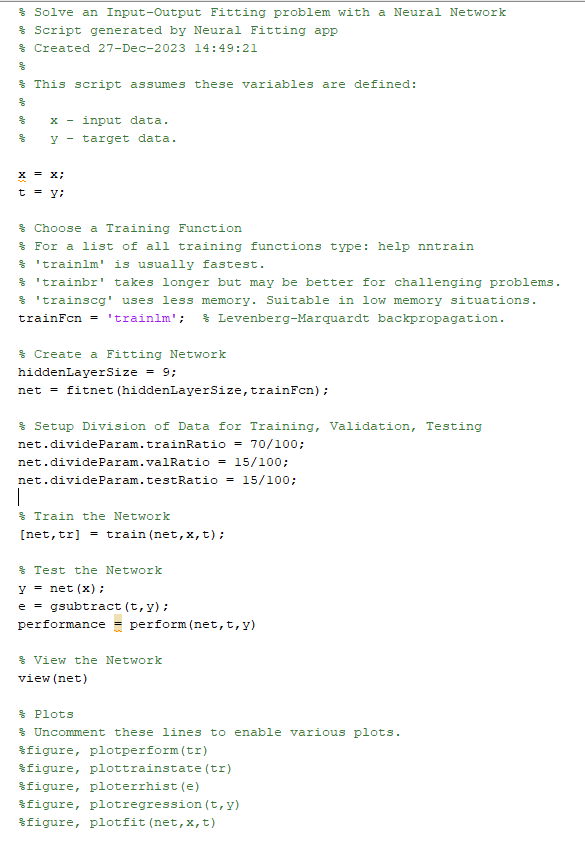


**Figure S4. MATLAB script of prediction neural network with nine hidden neurons**. The MATLAB script utilized Levenberg-Marquardt (LM) backpropagation and dataset is partitioned into three components: the training dataset, the validation dataset, and the test dataset, with a ratio of 70%, 15%, and 15%, respectively.


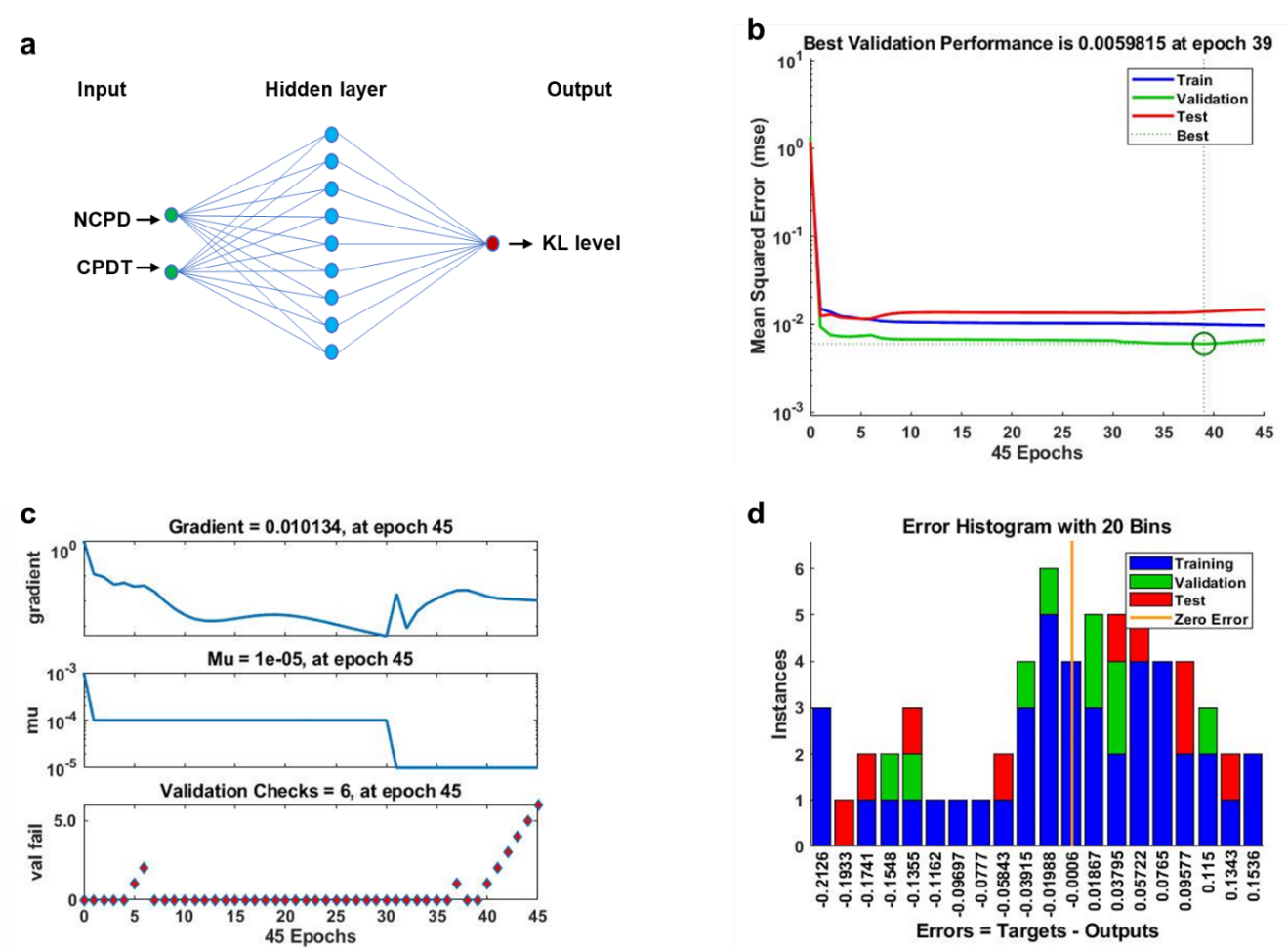


**Figure S5. The predictive backpropagation (BP) neural network.** (**a**) The structure of the BP ANN model. (**b**) The MSE represents the performance of the predictive module. (**c**) The model training states gradient, Mu, and validation fail statistics as a function of the number of epochs. (**d**) The error histogram of the trained neural network for the training, validation, and testing phases.


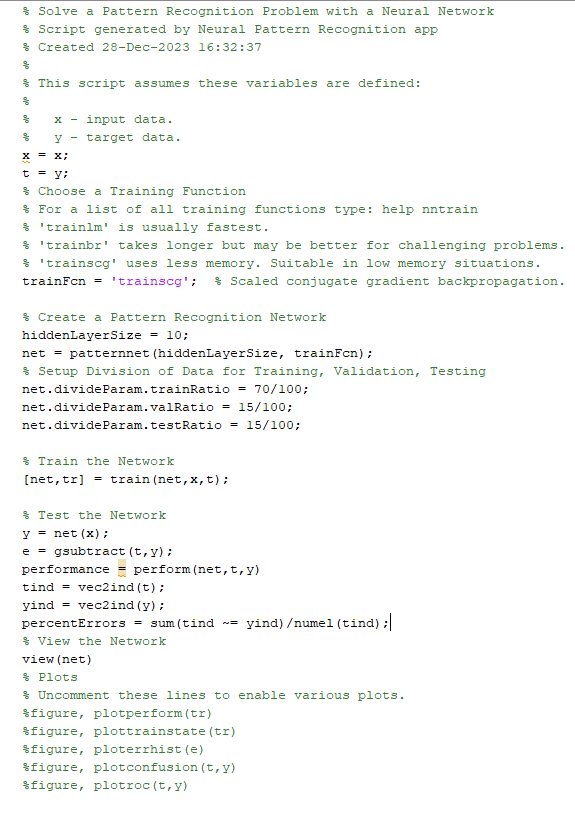


**Figure S6. MATLAB script of classification neural network with nine ten neurons**. The MATLAB script utilized Scaled Conjugate Gradient (SCG) backpropagation and dataset is partitioned into three components: the training dataset, the validation dataset, and the test dataset, with a ratio of 70%, 15%, and 15%, respectively.


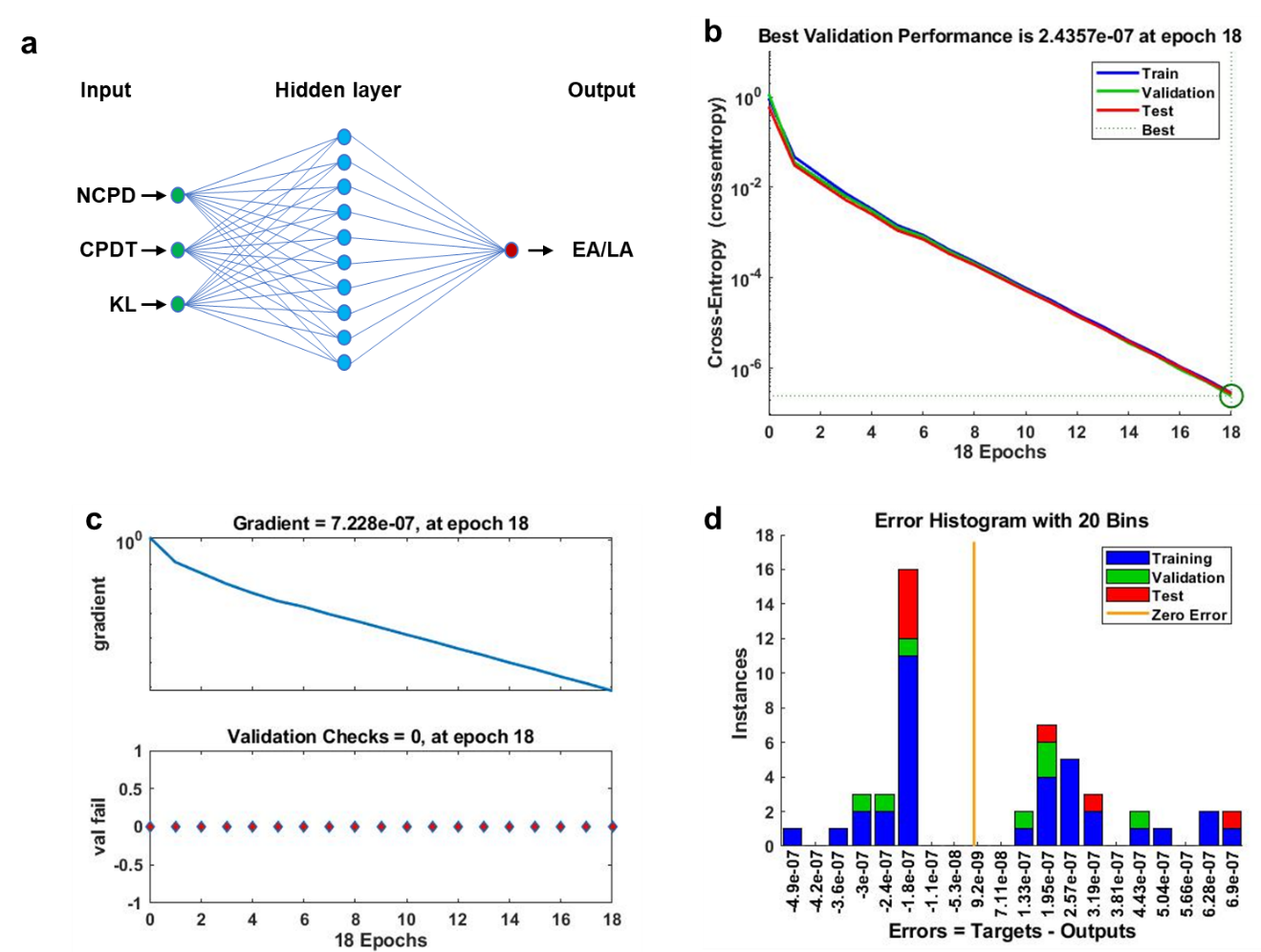


**Figure S7. The accuracy of the classification ANN model**. (**a**) The structure of proposed ANN classification model. (**b**) Cross-entropy represents the performance of the classification module of the EA- or LA-hNPCs. (**c**) The model training states with gradient and validation fail statistics as a function of the number of epochs. (**d**) The error histogram of the trained neural network for the training, validation, and testing phases.

References

1. Liu Y, Wang K, Yan ZY, Shen X, Yang X. Prediction of active ingredients in Salvia miltiorrhiza Bunge. based on soil elements and artificial neural network. *PeerJ*. 2022;10:e12726. doi:10.7717/peerj.12726

2. Woodward RB, Spanias JA, Hargrove LJ. User intent prediction with a scaled conjugate gradient trained artificial neural network for lower limb amputees using a powered prosthesis. *Annu Int Conf IEEE Eng Med Biol Soc*. Aug 2016;2016:6405-6408. doi:10.1109/EMBC.2016.7592194
