# Supplementary material for: Klotho mitigates intervertebral disc degeneration by regulating autophagy and energy metabolism
Source: Clin Transl Med. 2025 Jun 13;15(6):e70371. doi: 10.1002/ctm2.70371 (PMC12166123; doi:10.1002/ctm2.70371)
Supplement: Supplementary file 6 — Supporting Information [file CTM2-15-e70371-s007.docx]

**Method S5**

**BrdU cell proliferation assay**

Cell proliferation was determined by the BrdU Cell Proliferation Assay Kit (#6813, Cell Signaling Technology, USA) according to the manufacturer’s instructions. hNPCs (1x10^4^ cells/well) were seeded in a 96-well plate and incubated at 37°C for 72 h. Then, the BrdU solution was added to cells followed by an additional incubation for 6 h at 37°C. The incorporated BrdU was measured at 450 nm using a Victor Nivo Multi-plate reader (Perkin Elmer, USA).

**Method S6**

**Crystal violet assay**

Cells were cultivated at a given condition for the crystal violet proliferation assay for the indicated times. Cells were washed twice in 1xPBS and incubated with 10% formalin in 1xPBS for 20 min at room temperature. After removal of formalin, and 0.1% (w/v) crystal violet (#C0775-25G, Sigma) was added to the cells and further incubated for 20 min at room temperature. Crystal violet solution on the plates was thoroughly washed with drain water, followed by drying at room temperature. For quantification, 1% SDS was added to each well and incubated at room temperature for 30 min. The extracted solution in a fresh plate was quantified by measuring the OD at 590 nm under a Victor Nivo Multi-plate reader (Perkin Elmer, USA).

**Method S7**

**Senescence-associated β-galactosidase staining (SA-β-gal staining)**

The cellular senescence activity of hNPCs was determined using a senescence assay kit (#ENZ-KIT129, Enzo Life Sciences, Lausen, Switzerland) according to the manufacturer's instructions. First, hNPCs were seeded at 1x10^4^ cells/well in a 96-well plate and incubated for 72 h. Then hNPCs were lysed on ice with a lysis buffer and incubated at 4°C for 5 minutes after washing with 1x phosphate-buffered saline (PBS) buffer. The supernatant (50 µL) of cell lysates was transferred to a 96-well plate with 50 µL of freshly prepared Assay Buffer and further incubated at 37°C f at a light-free condition for 2 h. The reaction mixture (50 µL) was transferred to a 96-well plate, and the reaction was stopped by adding 200 µL of Stop solution. The fluorescence was determined at 360 nm (Excitation)/465 nm (Emission).

**Method S8**

**Measurement of Autophagy Flux**

In accordance with the manufacturer's protocol, the autophagic flux was evaluated using the CYTO-ID Autophagy detection kit (#ENZ-51031, Enzo Life Sciences, Lausen, Switzerland). The cells were cultivated in a 37 °C incubator with 5% carbon dioxide (CO_2_) for the duration of the experiment. The cells were washed twice with PBS at ambient temperature and then centrifuged at 400× g for 5 minutes without agitation. Following removing the supernatant, the cell pellets were reconstituted in 200 µL of PBS at ambient temperature for 20 minutes. Subsequently, 0.4 µL of Cyto-ID Green stain solution was introduced, and the cells were colored for 5 minutes at ambient temperature. Furthermore, 0.2 µL of Hoechst 33342 stain solution was added and incubated for 20 minutes. The cells were collected and stained with Cyto-ID Green fluorescent dye to quantify the autophagic flux. The dye was detected at an excitation wavelength of around 480 nm and an emission wavelength of around 530 nm.

**Method S9**

**Seahorse real-time cell metabolic analyses**

Cells were seeded on Seahorse XF96 Cell Culture microplates to confluence (40000 cells/well) and subjected to the metabolic profiling in two days sextuplicate after cell seeding. For the Cell Energy phenotype profile (CEP) and Mito stress tests (MST), the growth medium was replaced with Seahorse XF DMEM Base Medium (#103575-100, Agilent Technologies, pH adjusted to 7.4), supplemented with 10 mM D-glucose, 2 mM L-glutamine, and 1 mM sodium pyruvate. Cells were then cultured for 45 min in a CO_2_-free incubator at 37 °C. Oxygen consumption rates (OCR) were monitored at basal conditions and, after sequential injections of 2.5 µM oligomycin (Oligo) to block the mitochondrial ATP synthase, 1.25 µM FCCP to uncouple oxidative phosphorylation (OXPHOS) and 0.5 µM rotenone and antimycin A (Rot/AA) was used to inhibit mitochondrial respiration fully. For glycolysis stress tests (GST), the growth medium was replaced with Seahorse XF Base Medium (#103575-100, Agilent Technologies, pH adjusted to 7.4), supplemented with only two mM L-glutamine, and cells were cultured for 45 min in a CO_2_-free incubator at 37 °C. Extracellular acidification rate (ECAR) was monitored at basal conditions and after sequential injections of 10 mM D-glucose, 2.5 µM oligo, and 50 mM 2-deoxyglucose (2-DG), a glycolysis inhibitor. For glycolytic rate analysis (GRA), the medium was changed to Seahorse XF Base DMEM Medium (#103575-100, Agilent Technologies, pH adjusted to 7.4), supplemented with 10 mM glucose, 2 mM L-glutamine, 1 mM sodium pyruvate). Then, cells were cultured for 45 min in a CO_2_-free incubator at 37 °C, and proton efflux rate (PER) was recorded following injections with 0.5 µM Rot/AA and 50 mM 2-DG. OCR, ECAR, and PER measurements were performed in a 3 min mix and 3 min measure cycles at 37 °C on a Seahorse XFe96 Analyzer (Agilent Technologies). Wave software (Agilent Technologies) was used to analyze the datasets. OCR, ECAR, and PER were represented as pmol/min, mpH/min, and pmol/min, respectively, and normalized to the protein concentration of each well measured by BCA assay.

**Method S10**

**Mitochondrial superoxide (MitoSox) assay**

Mitochondrial superoxide radicals in hNPCs were measured with a Mitochondrial Superoxide Detection Kit (#ab219943, Abcam, Cambridge, United Kingdom) according to the manufacturer's protocols. Briefly, cells were seeded in triplicate (8,000 cells) into poly-L-lysine-coated 96-well microplates. Then the attached cells were stained with MitoROS 580 for 30 minutes at 37 °C. Superoxide radicals were determined using a fluorescence microplate reader (excitation/emission wavelength of 540/590 nm).

**Method S11**

**Interleukin 1 beta (IL-1β) measurement**

The cellular IL-1β level was determined by the ELISA assay (#ADI-900-130A, Enzo Life Sciences, Farmingdale, NY, USA) following the manufacturer’s instructions. Briefly, homogenate cell lysates (50 µL) were incubated with 100 µL human IL-1β biotin conjugate solution in human IL-1β antibody-coated wells (96-well strip-well plate) at room temperature for 2 h. A standard curve was calculated using a human IL-1β standard at different concentrations (0, 3.9, 7.8,15.6, 31.2, 62.5, 125 and 250 pg/mL). After washing, 100 µL streptavidin-peroxidase substrate solution was added into each well except for the chromogen blanks and incubated for 30 min at room temperature. The reaction was stopped with 100 µL stop solution in each well. The optical densities were measured at 450 nm using a microplate reader within 2 h after addition of the stop solution. The IL-1β concentrations were calculated with a standard curve using the GraphPad Prism software.

**Method S12**

**Western blotting**

Cells were collected and lysed with RIPA lysis and extraction buffer (#89900, Thermo Fisher Scientific) containing 1x Halt Protease and Phosphatase Inhibitor Cocktail (#78441, Thermo Fisher Scientific). The protein concentration of total cell lysates was determined using the Pierce BCA Protein Assay Kits (#89900, Thermo Fisher Scientific) according to the manufacturer’s instructions. The protein samples were separated on a SDS-PAGE gel and transferred to a PVDF membrane (GE Healthcare-Amersham Biosciences) using a semi-dry transfer system (Bio-Rad). The membrane was blocked for 45 min at room temperature with 5% skim milk in TBST. After incubation with primary antibodies (**Table S2**) overnight at 4 ^°^C with skim milk, the membrane was washed three times in TBST for 10 min each and then incubated with secondary antibodies (**Table S2**) in TBST for one hour. The membrane was subsequently washed three times with TBST for 10 min each and signal detected with Enhanced Chemiluminescence (ECL) detection system (#34080, Thermo Fisher Scientific). Proteins were quantified using ImageJ^®^ software (Version 1.53e, NIH, Bethesda, MD, USA). The graphical representations are the mean values (± SD) of at least three independent experiments.

**Table S2: List of antibodies**

| Antibodies | Target | Host | Clonality | Catalog # | Company |
| --- | --- | --- | --- | --- | --- |
| Primary  antibody | Klotho (KL) | Mouse | Monoclonal | sc-515942 | Santa Cruz |
|  | FGF-23 | Rabbit | Polyclonal | ab192497 | Abcam |
|  | COL2A1 | Rabbit | Polyclonal | ABP0074 | Abbkine |
|  | Aggrecan (AGCN) | Mouse | Monoclonal | sc-33695 | Santa Cruz |
|  | MMP-13 | Mouse | Monoclonal | sc-515284 | Santa Cruz |
|  | ADAMTS-5 | Rabbit | Polyclonal | ab182795 | Abcam |
|  | Cleaved caspase-3 | Rabbit | Polyclonal | 9661 | Cell Signaling |
|  | Caspase-3 | Rabbit | Polyclonal | 9662 | Cell Signaling |
|  | Bcl-2 | Mouse | Monoclonal | sc-7382 | Santa Cruz |
|  | p16 | Mouse | Monoclonal | sc-56330 | Santa Cruz |
|  | p21 | Mouse | Monoclonal | sc-6246 | Santa Cruz |
|  | p53 | Mouse | Monoclonal | sc-126 | Santa Cruz |
|  | LC3-I/II | Rabbit | Monoclonal | 12741 | Cell Signaling |
|  | p62 | Rabbit | Polyclonal | 5114 | Cell Signaling |
|  | Beclin-1 | Rabbit | Monoclonal | 3495 | Cell Signaling |
|  | β-actin | Mouse | Monoclonal | sc-47778 | Santa Cruz |
| Secondary antibody | Mouse IgG | Goat | Polyclonal | STAR117P | Bio-Rad |
|  | Rabbit IgG | Goat | Polyclonal | STAR208P | Bio-Rad |

**Note S7**

The proliferative capability of hNPCs during *in vitro* cell culture was assessed through examination of cell morphology using phase contrast microscopy, BrdU incorporation, and clonogenic ability using the crystal violet test in EA- and LA-NPCs. Distinct alterations in cell morphology were observed between EA-hNPCs and LA-hNPCs, with LA-NPCs exhibiting a shift from a spindle form to a slightly enlarged, flattened, irregular shape, indicative of reduced cell growth rates, reflecting cellular senescence (**Figure 2e**). To evaluate premature cellular senescence, SA-β-gal activity and the expression level of senescence proteins, namely p16, p21, and p53 were determined. Results indicated that the LA-hNPCs exhibited higher SA-β-gal activity than the EA-hNPCs (p<0.0001, **Figure 2f**). Correspondingly, the levels of cellular senescence markers, including p16, p21, and p53, were significantly elevated enhanced in the LA-hNPCs compared to the EA-hNPCs (p<0.01, p<0.001, **Figure 2g**). These findings suggest that hNPCs undergo premature senescence in LA during continuous *in vitro* expansion. Notably, LA-hNPCs displayed significantly lower proliferation rates, evidenced by the reduced clonogenic cell growth rate (p<0001, **Figure 2h**), further supporting the occurrence of cellular senescence. As expected, LA-hNPCs demonstrated markedly diminished proliferation rates, with lower incorporation of BrdU compared to EA-hNPCs (p<0.0001, **Figure 2i**). Importantly, LA-hNPCs displayed increased cleaved caspase-3 and decreased Bcl-2 level compared to EA-hNPCs (p<0.01, **Figure 2j**).

Senescent cells release senescence-associated secretory proteins (SASP), such as IL6 and IL1β, known to contribute to an inflammatory environment in aging-related disorders ^1,2^. Notably, IL1β was higher in LA-hNPCs compared to EA-hNPCs (p<0.001, **Figure 2k**). Accordingly, analysis of ECM-related proteins, including COL2A1, aggrecan, MMP-13, and ADAMTS-5, revealed that LA-hNPCs exhibited reduced COL2A1, AGCN, and increased MMP-13 and ADAMTS-3 compared with EA-hNPCs (p<0.05, p<0.01, p<0.0001, **Figure 2l**).

We further investigated whether the involvement of autophagy pathways correlates with an increase in cell senescence, given their potential role in the development of IVDD phenotypes. The aforementioned results underscored the expression of IVVD phenotypes in LA-hNPCs, leading to apoptosis and ECM imbalance during continuous *in vitro* expansion. Analysis of autophagy markers, often used as indicators of autophagy flux, revealed that LC3, and Beclin-1 levels were decreased, while p62 was upregulated in LA-hNPCs compared with EA-hNPCs (p<0.01, **Figure 2m**). Interestingly, mitochondrial ROS levels were higher in LA-hNPCs than in EA-hNPCs (p<0.001), suggesting a potential association between oxidative stress and cellular senescence in these cells (**Figure 2n, Figure S8a**).

Mitochondrial biogenesis has been implicated to inducing cellular senescence, partially through the elevation of ROS-mediated molecular damage ^3-5^. Consequently, we explored whether mitochondrial respiration, a primary source of ROS generation, was increased in senescent hNPCs. Using a Seahorse extracellular flux analyzer, we performed bioenergetic phenotyping of hNPCs, assessing the glycolytic and oxidative potential of senescent cells based on the Agilent Seahorse XF cell energy phenotype (CEP) (**Figure S8b**). A discernible shift in bioenergetics profiling was observed in LA-hNPCs. Indicating a transition in cell glucose metabolism from a quiescent state toward an energetic phase (**Figure 2o**). The metabolic potential of LA-hNPCs appeared to be heightened under basal or energy stress conditions compared to EA-hNPCs (p< 0.05, p<0.0001, **Figure 4p-r**), suggesting that the LA-hNPCs rely more on cellular energy metabolism than EA-hNPCs.

To profile energy metabolism in replicative senescent cells, we examined mitochondrial respiration in the Seahorse system using the mitochondrial stress test (MST) for oxygen consumption rate (OCR) (**Figure S8c**), glycolytic function using the glycolysis stress test (GST) for extracellular acidification rate (ECAR) (**Figure S8d**) and glycolytic activity using the glycolytic stress test assay (GTA) for proton efflux rate (PER) (**Figure S8e**) in both EA- and LA-hNPCs.

The MST evaluated mitochondrial respiration by OCR under basal conditions and following the addition of specific inhibitors of the respiratory chain (Rote, AA, and oligo to inhibit complex I, III, and V, respectively) or uncoupler of the proton gradient (FCCP) (**Figure 2s, Figure S8c**). Interestingly, an elevated OCR was observed in LA-hNPCs compared to EA-hNPCs (**Figure 2s**). The ECAR value in LA-hNPCs showed a significant rise, serving as an indirect indication of glycolytic ability according to the MST (Fig. 2t). Derived OCR values provided insights into basal respiration (BR), maximal respiration (MR), proton leak (PL), mitochondrial ATP production, and respiratory spare capacity (RSC) (**Figure 2v**). In comparison to EA-hNPCs, LA-hNPCs exhibited a significant increase in BR, MR, PL, mitochondrial ATP production, and RSC (p<0.001, p<0.0001, **Figure 2v**). LA-hNPCs displayed a significant increase in OCR compared to EA-hNPCs under basal conditions and in response to ATP synthase inhibitor (Oligomycin), complex I inhibitor (Rotenone) or complex III inhibitor (Antimycin A), and FCCP uncoupler (p<0.05, p<0.0001, **Figure 2u**), indicating their ability to enhance oxidative phosphorylation (OXPHOS) in response to mitochondrial respiration.

Furthermore, the Seahorse GST analysis assesses glycolytic pathway capacity under various conditions, demonstrating increased ECAR in LA-hNPCs compared to EA-hNPCs (**Figure 2w, Figure S8d**). Calculations based on ECAR values revealed higher basal glycolysis (BG) and a significant increase in glycolytic capacity (GC) and glycolytic reserve (GR) in LA-hNPCs compared to EA-hNPCs (p<0.0001, **Figure 2x**), indicating their ability to boost glycolytic flux in response to increased energy demands.

In addition, the glycolytic rate assay (GRA) provided a quantifiable measurement of glycolysis under physiological media conditions, indicating increased PER in LA-hNPCs compared to EA-hNPCs (**Figure 2y, Figure S8e)**. These PER values were used to calculate basal respiration (BR), basal PER (BPER), compensatory glycolysis (CG), and post-2-DG acidification (P2DA), all of which were increased in LA-hNPCs compared to EA-hNPCs (p<0.001, p<0.0001, **Figure 2z**).

In summary, these findings reveal an augmentation in oxidative glucose metabolism as a distinctive feature of replicative senescent cells.

*
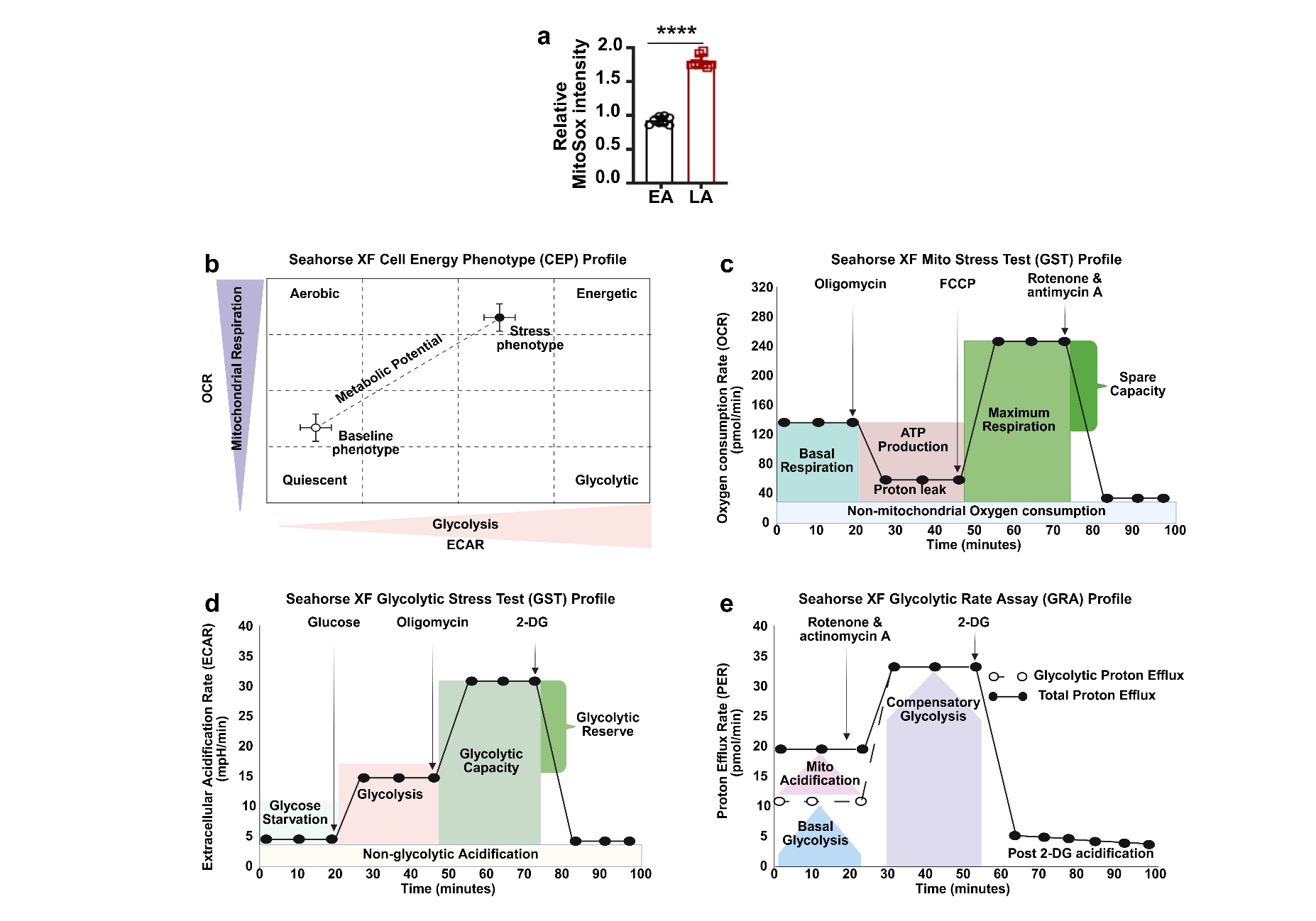
*

**Figure S8: The bioenergetics profiling of EA and LA-hNPCs.** (**a**) The measurement of mitochondrial ROS in EA- and LA-hNPCs. A typical graph of the Agilent Seahorse XF Cell Energy Phenotype profile. (**b**) A typical graph of the Agilent Seahorse XF Mito stress test (MST). (**c**) A typical graph of the Agilent Seahorse XF Glycolytic stress test (GST). (d) A typical graph of the Agilent Seahorse XF Glycolytic rate assay (GRT).

**References**

1. Sabbatinelli J, Prattichizzo F, Olivieri F, Procopio AD, Rippo MR, Giuliani A. Where Metabolism Meets Senescence: Focus on Endothelial Cells. *Front Physiol*. 2019;10:1523. doi:10.3389/fphys.2019.01523

2. Coppe JP, Patil CK, Rodier F, et al. Senescence-associated secretory phenotypes reveal cell-nonautonomous functions of oncogenic RAS and the p53 tumor suppressor. *PLoS Biol*. Dec 2 2008;6(12):2853-68. doi:10.1371/journal.pbio.0060301

3. Summer R, Shaghaghi H, Schriner D, et al. Activation of the mTORC1/PGC-1 axis promotes mitochondrial biogenesis and induces cellular senescence in the lung epithelium. *Am J Physiol Lung Cell Mol Physiol*. Jun 1 2019;316(6):L1049-L1060. doi:10.1152/ajplung.00244.2018

4. Correia-Melo C, Marques FD, Anderson R, et al. Mitochondria are required for pro-ageing features of the senescent phenotype. *EMBO J*. Apr 1 2016;35(7):724-42. doi:10.15252/embj.201592862

5. Passos JF, Saretzki G, Ahmed S, et al. Mitochondrial dysfunction accounts for the stochastic heterogeneity in telomere-dependent senescence. *PLoS Biol*. May 2007;5(5):e110. doi:10.1371/journal.pbio.0050110
