# Supplementary material for: Klotho mitigates intervertebral disc degeneration by regulating autophagy and energy metabolism
Source: Clin Transl Med. 2025 Jun 13;15(6):e70371. doi: 10.1002/ctm2.70371 (PMC12166123; doi:10.1002/ctm2.70371)
Supplement: Supplementary file 7 — Supporting Information [file CTM2-15-e70371-s008.docx]

**Method S13**

**siRNA**

Cells were transfected with KL siRNA (siKL; #sc-43883, Santa Cruz,) and siRNA-noncoding control (siNC; #sc-37007, Santa Cruz) using Lipofectamine 3000 (Invitrogen, Thermo Fisher Scientific) as described by the manufacturer’s instruction. hNPCs were transfected with NPCM (without supplement) containing 75 pmol of si-KL/si-NC at approximately 60–70% confluency for 6 hours. Then cells were further incubated with the same fresh medium for 24 hours. After cell lysis, cell extracts were subjected to western blotting analysis to confirm the expression of indicated proteins.

**Note S8**

To investigate the causal relationship between the shift towards senescence and the loss of KL in LA-hNPCs, we employed siRNA-mediated knockdown of KL in EA-hNPCs and verified the efficiency using Western blot analysis (**Figure 3a**). The colonies formed by KL knockdown hNPCs appeared smaller and less dense compared to control cells (**Figure 3b**). Quantification of crystal violet released from cells on each plate at 590 nm revealed a significant reduction in the growth rate or clonogenic activity of KL-deficient cells compared to control cells (p<0.0001, **Figure 3c**), indicating a diminished proliferative ability.

Furthermore, KL-knockdown hNPCs exhibited higher mitochondrial ROS production, as indicated by an increase in mitoSOX levels compared to control cells (p<0.0001, **Figure 3d**). The heightened mitochondrial superoxide production suggested a connection between KL and the mitochondrial functions of these cells. We then investigated various parameters of mitochondrial respiration in KL-deficient hNPCs using MST, GST, and GRA in the Seahorse system (**Figure. 3d-i**). In comparison to the control, KL-deficient hNPCs displayed higher OCR, ECAR, and PER in MST, GST, and GRA, respectively (**Figure 3d, f, g**). KL-knockdown hNPCs showed a significant increase in BR, MR, mitochondrial ATP production, and RSC (p<0.0001, **Figure 3e**). While there was a mild to moderate increase in OCR on PL, it did not reach statistically significant (p=0.0746, **Figure 3e**). The results indicated higher glycolysis in KL-deficient hNPCs, as evidenced by elevated BG and a significant increase in GC and GR (p<0.0001, **Figure 3h**). Additionally, KL-deficient hNPCs exhibited increased BG, BPER, and CG (p<0.0001, **Figure 3i**).

Subsequently, we examined whether KL knockdown could alter autophagic flux in hNPCs. Indeed, KL knockdown reduced overall autophagy by decreasing the expression of LC3II/I and Beclin-1 and increasing p62 level (**Figure 3j, k**). Similar to high-passage cells, KL-depleted or siKL-treated hNPCs underwent accelerated cellular senescence, as evidenced by higher SA-β-gal activity (**Figure 3l**). Correspondingly, the levels of cellular senescence markers, p16, p21, and p53, were upregulated in the siKL-treated cells (**Figure 3m**). In addition, KL knockdown exhibited reduced COL2A1 and AGCN and increased MMP-13 and ADAMTS-3, along with high IL1B compared to non-targeting siRNA-expressing cells (**Figure 2n,o**). It also displayed lower proliferation rates, as evidenced by incorporating less BrdU into KL-depleted cells than control cells (**Fig. 2p**). Moreover, the knockdown of KL showed increased cleaved caspase-3 and decreased Bcl-2 activity compared to control cells (**Figure 2q)**.
