# Supplementary material for: Klotho mitigates intervertebral disc degeneration by regulating autophagy and energy metabolism
Source: Clin Transl Med. 2025 Jun 13;15(6):e70371. doi: 10.1002/ctm2.70371 (PMC12166123; doi:10.1002/ctm2.70371)
Supplement: Supplementary file 8 — Supporting Information [file CTM2-15-e70371-s001.docx]

**Method S14**

**Human recombinant Klotho protein**

The human recombinant KL (rKL) protein was purchased from Bio-Techne R&D Systems (#34–981a.a., 5334-KL-025, R&D Systems, USA). The recombinant protein samples were separated on an SDS-PAGE gel and transferred to the PVDF membrane (GE Healthcare-Amersham Biosciences) using a semi-dry transfer system (Bio-Rad). Proteins on the PVDF membranes were visualized by a brief incubation in the Ponceau S or Coomassie solution (PageBlue Protein Staining Solution, Thermo Scientific; application on dried membranes). The optimal dose for subsequent experiments were determined by incubating cells in a 96-well plate for 24 h, 48, and 72 h with varying doses of rKL (50, 100, 200,400,500, 800, and 1000 ng/mL). The rKL concentration (400 ng/mL), indicating about 30 to 35 % cell viability of LA-hNPCs, was used for the subsequent modeling experiments.

**Method 15**

**Three-dimensional (3D) spheroidal**

The *in vitro* 3D spheroids were generated using a modified hanging drop and method to study the growth dynamics of EA and LA NPCs, as well as LA NPCs treated with rKL. NPCs were cultured at a density of 2×10^6^ cells/ml in 20 µl drops on the inverted lid of a 60 mm petri dish containing 4 to 5 ml of PBS in bottom to maintain humidity. After incubation for 72 h, the aggregate cell sheets were transferred to a poly 2-hydroxyethyl methacrylate (Poly-HEMA)-coated plate containing a complete medium. The cultures were maintained in a humidified incubator at 37°C with 5% CO₂. After an additional 72 h, when uniform spheroids had formed, they were prepared for microscopic observation. Observations and measurements of spheroid diameter were conducted at the following time points: day 1 (initial observation), and subsequently on days 4, 11, 17, and 24. The diameter of each spheroid was measured using ImageJ software, providing quantitative data on spheroid growth over time.

**Note S9**

Recombinant KL was identified through a Coomassie and Ponceau S-stained PVDF membrane following electrophoresis on a 10 % polyacrylamide gel (**Figure S9a**). The cell viability of rKL was assessed at 24, 48, and 72 h revealing enhanced cell viability at 200 and 400 ng/mL rKL after incubation for 72 h (**Figure S9b-d**). The colony formation assay (CFA) indicated an elevated cell growth rate with rKL treatment (**Figure 4a**). Western blot data demonstrated a significant increase in KL and FGF-23 protein expression upon rKL treatment (**Figure 4a**). Treatment with rKL (200 and 400 ng/mL) effectively restored mitochondrial respiration, balancing glycolysis and OXPHOS to achieve mitochondrial homeostasis (Figure 4b-g). Specifically, rKL treatment (200 or 400 ng/mL) led to decreased OCR, ECAR, and PER in MST, GST, and GRA, respectively (**Figure 4b, d, f**). Furthermore, rKL significantly reduced BR, MR, PL, mitochondrial ATP production, and RSC in LA-NPCs (p<0.05, p<0.01, p<0.0001, **Figure 4c**). The results indicated a gradual suppression of higher glycolysis in rKL-treated hNPCs, as evidenced by lowered BG, GC, and GR (p<0.05, p<0.001, p<0.0001, **Figure 4e**). Additionally, rKL-treated hNPCs exhibited decreased BR, BPER, and CG (p<0.001, p<0.0001, **Figure 4g**).

**Note S10**

In our 3D cell culture experiment, we observed distinct developmental patterns between EA and LA spheroids (**Figure 4h**). The EA spheroids showed continuous growth throughout the experiment, with size measurements taken on days 1, 4, 11, 17, and 24 reflecting a consistent increase. In contrast, the LA spheroids did not exhibit any significant growth during the same period, indicating a cessation of their growth potential. Remarkably, treating LA spheroids with rKL led to a substantial increase in growth, suggesting that recombinant KL plays a crucial role in reactivating cellular proliferation pathways in aged spheroids. This unique response highlights the potential of recombinant KL as a therapeutic agent for regulating spheroid growth dynamics, particularly when natural growth processes have stalled.

Additionally, Mitosox Red fluorescence intensity, which indicates mitochondrial reactive oxygen species (ROS) levels, was significantly higher in LA spheroids, reflecting increased oxidative stress in these aged cells (**Figure 4i, Figure S10a**). However, rKL treatment significantly reduced Mitosox Red fluorescence intensity, suggesting a potential antioxidative effect of rKL that may help restore growth in LA spheroids. This response underscores the potential of rKL as a therapeutic agent in managing spheroid development dynamics and reducing oxidative stress, especially when normal growth processes have been disrupted.

Moreover, western blotting results showed that rKL treatment significantly enhanced the expression of KL and LC3 II, an autophagy marker, while reducing the expression of p62, a protein associated with autophagic degradation (**Figure 4j, Figure S10b**). These findings suggest that rKL not only promotes growth and reduces oxidative stress in LA spheroids but also enhances autophagic processes. This presents a multifaceted therapeutic potential for rKL in regulating spheroid growth dynamics and maintaining cellular homeostasis, particularly in situations where natural growth processes have ceased.

**Note S11**

To explore the role and regulatory mechanism of rKL in hNPCs, we treated chloroquine (CQ) to inhibit autophagic flux, aiming to determine whether rKL enhances cell proliferation and mitochondrial homeostasis through autophagic regulation in LA-hNPCs. The results revealed that co-treatment of rKL and CQ significantly impeded the autophagy-mediated protective activity of rKL in LA-hNPCs (**Figure 4k-u**). In particular, co-treatment of rKL and CQ reduced rKL-induced autophagy activity by decreasing the expression of LC3 and Beclin-1 and increasing p62 proteins in LA-hNPCs (**Figure 4k)**.

Also, CQ effectively blocked the rKL-mediated reduction of mitochondrial ROS by increasing mitoSOX production in LA-hNPCs (**Figure 4l**). Significantly, mitochondrial superoxide production was increased in rKL+CQ compared to only rKL-treated LA-hNPCs (p< 0.0229, **Figure 4l**). Furthermore, co-treatment of CQ and rKL disrupted the rKL-mediated mitochondrial homeostasis by influencing OCR, ECAR, and PER in LA-hNPCs (**Figure 4m-o).** Basal respiration, ATP production, and spare respiratory capacity were significantly elevated in rKL+CQ co-treated cells compared to only rKL-treated LA-hNPCs (p< 0.001, p<0.0132, and p<0.0325, **Figure S11a)**. Neither maximal respiration nor proton leak significantly affected maximal respiration (p > 0.0125, **Figure S11a**). Glycolytic capacity was significantly increased in rKL+CQ compared to only rKL-treated LA-hNPCs (p< 0.0001, **Figure S11b)**. Compensatory glycolysis was significantly increased in rKL+CQ compared to only rKL-treated LA-hNPCs (p< 0.0001, **Figure S12c)**.

While rKL-treated LA-hNPCs exhibited higher proliferation rates than control cells, this effect was blocked by CQ co-treatment (**Figure 4r**). The co-treatment of CQ and KL resulted in increased cleaved caspase-3 and decreased Bcl-2 activity compared to only rKL-treated LA-hHPC cells (**Figure 4s)**.

Additionally, blocking autophagy by CQ significantly increased SA-β-gal activity, which was reduced by rKL treatment (**Figure 4p**). Similarly, co-treatment of CQ and rKL significantly increased senescence markers, p16, p21, and p53, which were facilitated by rKL single treatment (**Figure 4q**). Furthermore, the pro-inflammatory gene IL1β was found to be elevated in co-treated cells compared to single rKL-treated LA-hNPCs (**Figure 4t**). The inhibition of autophagy by CQ reduced rKL-induced ECM secretion by decreasing induced COL2A1 and aggrecan and increasing MMP-13 and ADAMTS-5 (**Figure 4u**).


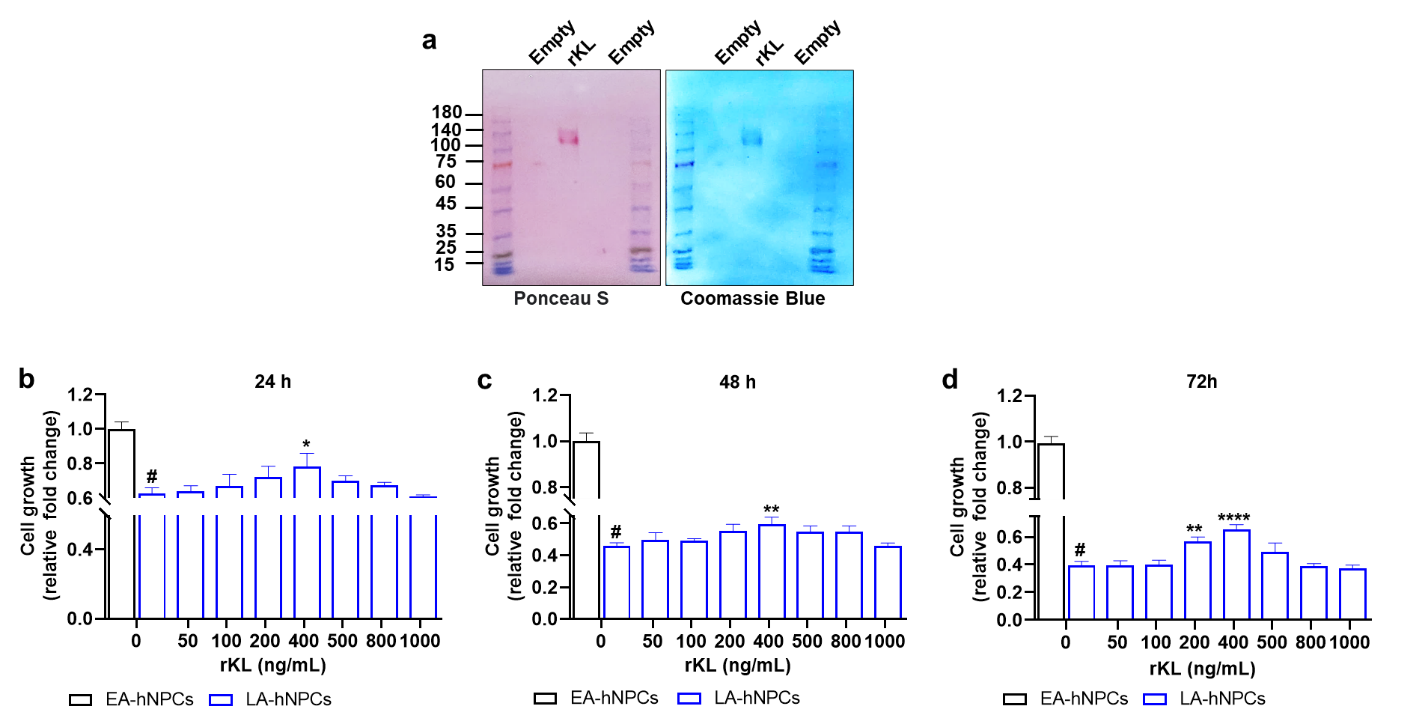


**Figure S9. Effective dose measurement of rKL protein.** (**a**) Coomassie (Left) and Ponceau S (right) stained PVDF membrane for rKL idendification.(**b, c, d**) The cell viability of different dose of rKL was measured at 24, 48, and 72 h. Values were represented as mean ± SD and statistical significance was determined using ordinary one-way ANOVA with Tukey’s multiple comparisons in **b**, **c**, **d**. ^#^ p < 0.0001 (EA-hNPCs vs LA-hNPCs at 0 ng/mL of rKL) and * p < 0.05, *** p < 0.001, **** p < 0.0001 (0 ng/mL vs 50-1000 ng/mL of rKL in LA-hNPCs ) considered as significantly different.

**
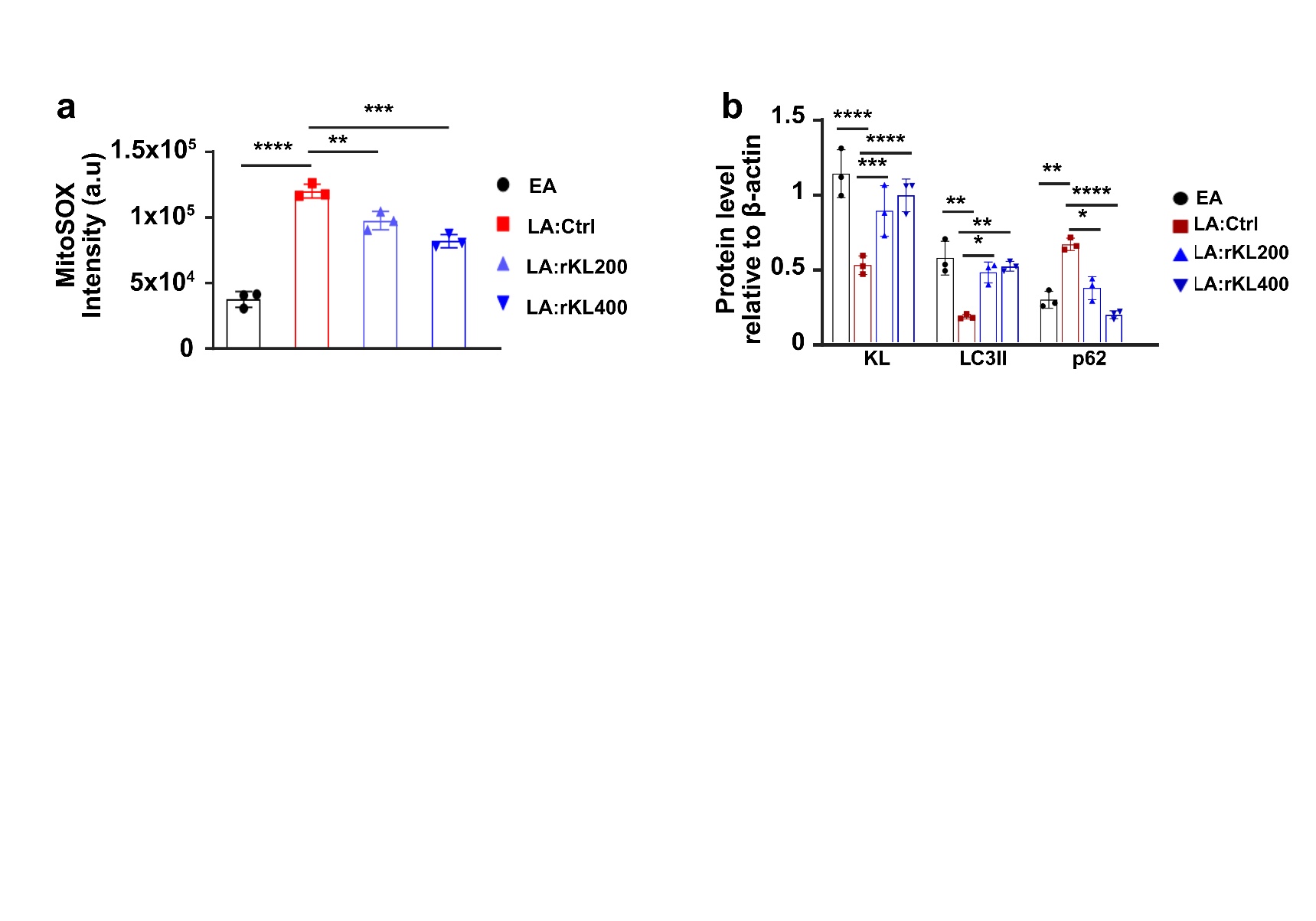
**

**Figure S10. Mitosox Red fluorescence intensity analysis.** (**a**) Treatment with rKL significantly reduced Mitosox Red fluorescence intensity, suggesting an antioxidative effect of rKL. (**b**) The normalized Western blot data revealed that rKL treatment significantly increased the expression of Klotho (KL) and LC3 II, an autophagy marker, while reducing the expression of p62, associated with autophagic degradation. Values were represented as mean ± SD and statistical significance was determined using ordinary one-way ANOVA with Tukey’s multiple comparisons in two-way ANOVA with Tukey’s multiple comparisons in **b**. * p < 0.05, ** p < 0.01, *** p < 0.001, and **** p < 0.0001 considered as significantly different.


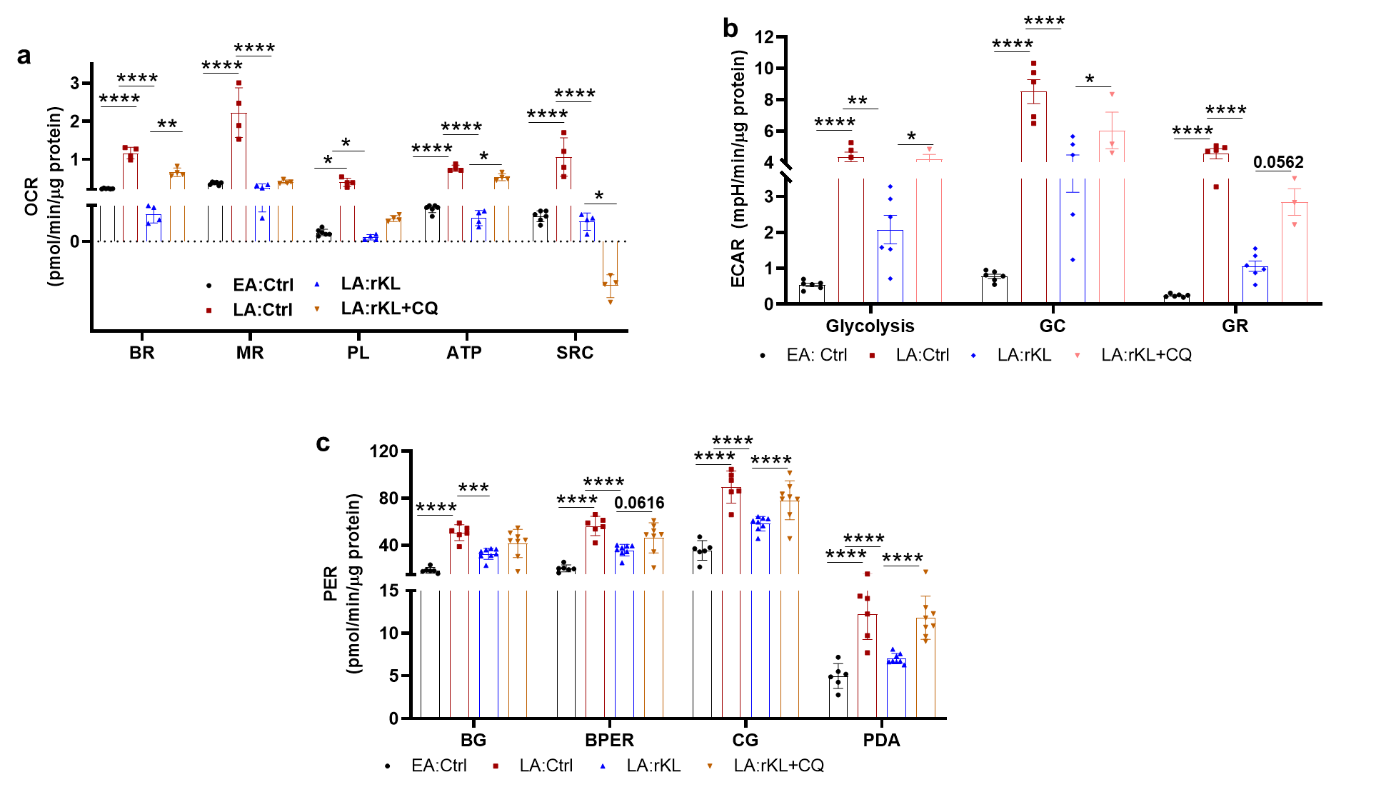
**Figure S11. rKL-induced autophagy and mitochondrial respiration of hNPCs in LA.** (a) The measurement of different parameters, including basal respiration (BR) and maximum respiration (MR), proton leak (PL), ATP production, and spare respiratory capacity (SRC) of LA-hNPCs after rKL+CQ treatment. (b) The measurement of different parameters, including glycolysis, glycolytic capacity (GC), and glycolytic reserve (GR) of LA-hNPCs after rKL+CQ treatment. (c) The measurement of different parameters, including basal glycolysis (BG), basal PER (BPER), compensatory glycolysis (CG), and post-2-DG acidification (PDA). Values were represented as mean ± SD and statistical significance was determined using two-way ANOVA with Tukey’s multiple comparisons. * p < 0.05, ** p < 0.01, *** p < 0.001, and **** p < 0.0001 considered as significantly different.
