# Supplementary material for: Klotho mitigates intervertebral disc degeneration by regulating autophagy and energy metabolism
Source: Clin Transl Med. 2025 Jun 13;15(6):e70371. doi: 10.1002/ctm2.70371 (PMC12166123; doi:10.1002/ctm2.70371)
Supplement: Supplementary file 9 — Supporting Information [file CTM2-15-e70371-s006.docx]

**Note S12**

Intervertebral disc degeneration is a multifaceted process involving age-related changes and tissue damage resulting from various stressors ^1^. Notably, cellular senescence of NPCs has emerged as a significant factor in intervertebral disc aging and IVDD progression ^2-4^. Senescent cells are implicated in tissue pathology, diminishing regenerative capabilities, promoting apoptosis, and contributing to ECM degradation through the manipulation of various effector proteins ^5,6^. This study aims to unravel the mechanism underlying cellular senescence in hNPCs by conducting assays to assess changes in cell proliferation, bioenergetics alteration, autophagy flux, mitochondrial ROS generation, spheroidal formation, apoptosis, and ECM gene expression during in-vitro aging.

Senescent cells exhibit decreased activation of the KL/FGF-23 axis, correlating with reduced cell proliferation and autophagy flux, which is accompanied by increased mitochondrial malfunction and oxidative phosphorylation. Recognizing changes in energy metabolism as key contributors to various diseases. Remarkable strides have been taken in delineating potential therapeutic targets in the field of aging research, with a particular focus on metabolic intermediates ^7-9^.

The Agilent Seahorse XF technology stands out as a crucial tool, offering essential functional data from live cells. This technology enables the identification of promising gene and protein targets, allowing for the assessment of their roles in aging cell proliferation, adaptability, and survival. Uncovering metabolic vulnerabilities in aging cells involve evaluating bioenergetics phenotype, cellular ATP production rate, and mitochondrial and glycolytic activity to delineate age-related changes. A fundamental step in comprehending biological aging is unveiling the metabolic and bioenergetics signature of the cell, as well as understanding the OCR/ECAR ratio. The cell energy phenotype analysis provides a comprehensive evaluation of bioenergetics balance, contributing significantly to advancing our understanding of cellular aging.

In this study we identified significant metabolic alteration in late-age hNPCs, particularly in higher-passage cells displaying a distinct metabolic profile linked to mitochondrial genetic and functional impairments. These impairments hindered mitochondrial biogenesis, leading to a metabolic shift from quiescent to energetics, indicative of cellular aging. The findings suggest that metabolic phenotypes could serve as indicators of cellular aging, and modulating altered metabolism could cell growth.

Senescent cells exhibit a metabolic shift characterized by heightened glycolysis, mitochondrial activity, and mitochondrial damage due to increased mitochondrial ROS generation. Based on the current findings, this shift was confirmed by the Mito stress test, demonstrating that senescent hNPCs exhibited a higher oxygen consumption rate than control cells. The glycolytic stress and rate assay further revealed increased glycolysis, PER, and lactate generation in senescent cells. These results contribute to the growing literature linking autophagy manipulation to IVDD pathogenesis ^10-13^, and decreased KL expression by NPCs after IVD damage ^14,15^. Consequently, targeting the autophagy pathway, KL/FGF-23 axis, or delivering rKL to the NP cells emerges as a potential strategy to delay or prevent the onset of senescence-associated IVDD.

While recognizing the potential benefits of autophagy in IVDD treatment, the study acknowledges conflicting findings from preclinical research using autophagy activators ^16-19^. Indeed, both macro-autophagy and chaperone-mediated autophagy are upregulated in the rat nucleus pulposus with age, and endoplasmic reticulum stress-mediated autophagy causes IVDD ^20-22^. Variability in outcomes may stem from different autophagy pathways. Addressing these challenges, we suggest targeting molecules upstream of autophagy activation, with anti-aging factor like KL emerging as potential therapeutic targets. Recent studies corroborate the decline in KL expression in degenerative discs, supporting the notion that restoring KL expression could counter SASP protein release, maintaining NPC phenotype and survival ^14,15^. Our research builds upon existing knowledge by focusing on the role of Klotho in regulating energy metabolism and autophagy, and its impact on cellular senescence and extracellular matrix balance in nucleus pulposus cells, without specifically targeting the Rac1/PAK1/MMP2 axis as earlier studies have emphasized. Moreover, unlike previous research that targets nucleus pulposus progenitor cells using KL circRNA, our study investigates the direct effects of recombinant Klotho on NPCs. These distinctions provide a fresh perspective on the therapeutic potential of KL, showcasing its complex role in cellular processes.

In the study, we suggest that KL-induced autophagy plays a crucial role during hNPCs aging (**Figure S12**), showcasing the interconnection of mitochondrial biogenesis, cellular senescence, autophagy, and KL expression. These findings support the hypothesis that KL deficiency is not only a biomarker but also a significant pathogenic component in IVDD development. Furthermore, rKL protein replacement emerges as a practical, safe, and effective approach to prevent or treat IVDD, offering potential applications in other age-related diseases like osteoarthritis. Importantly, rKL treatment demonstrates preventive effects on IVDD after cell senescence and protects IVD cells from mitochondrial dysfunction.


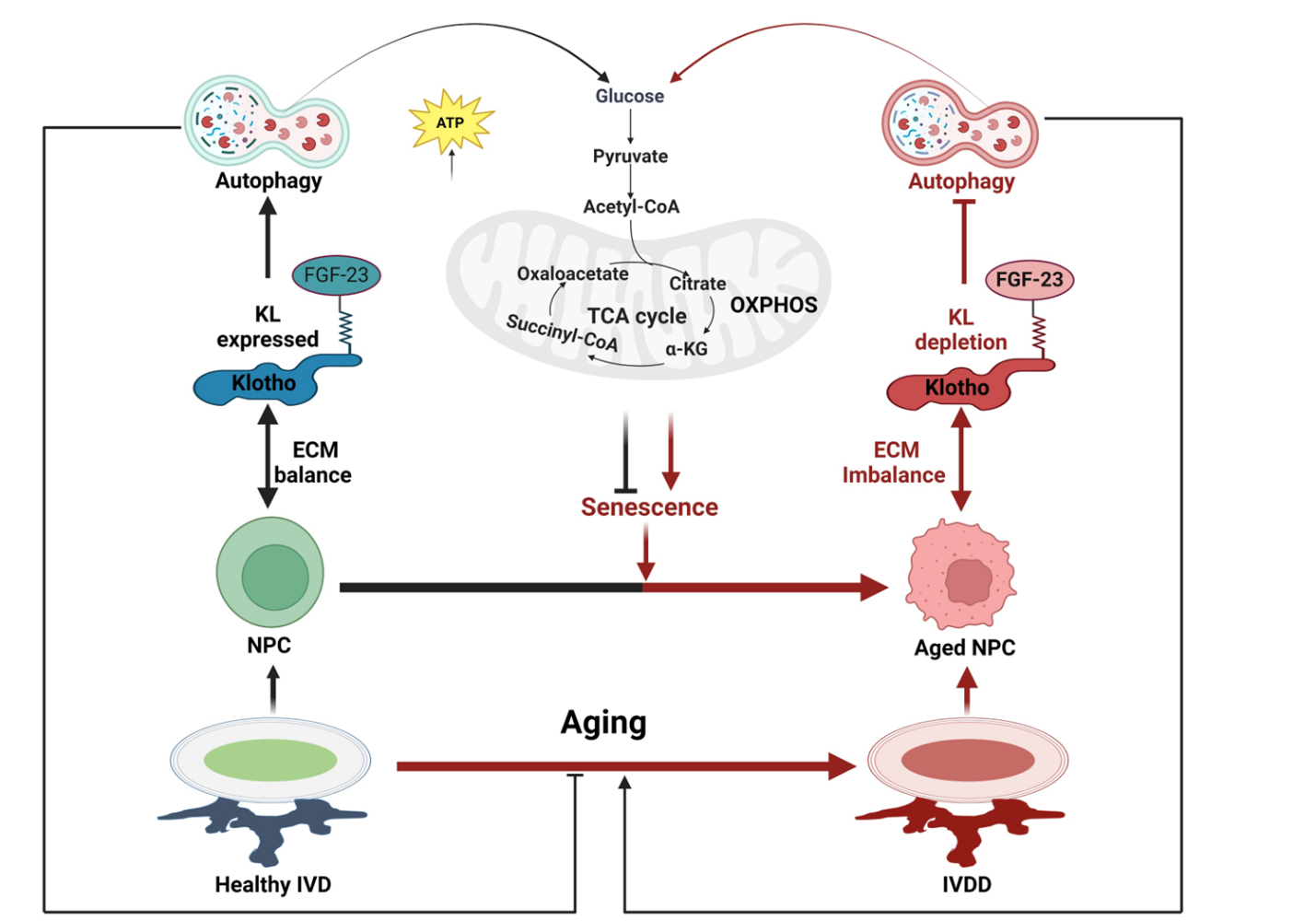


**Figure S12: The proposed mechanism of KL-induced autophagy in hNPCs aging.**

**References**

1. Wang F, Cai F, Shi R, Wang XH, Wu XT. Aging and age related stresses: a senescence mechanism of intervertebral disc degeneration. *Osteoarthritis Cartilage*. Mar 2016;24(3):398-408. doi:10.1016/j.joca.2015.09.019

2. Wang F, Zheng C, Wu X. [Research progress of cellular senescence and senescent secretary phenotype in intervertebral disc degeneration]. *Zhongguo Xiu Fu Chong Jian Wai Ke Za Zhi*. Dec 2012;26(12):1448-52.

3. Feng C, Liu H, Yang M, Zhang Y, Huang B, Zhou Y. Disc cell senescence in intervertebral disc degeneration: Causes and molecular pathways. *Cell Cycle*. Jul 2 2016;15(13):1674-84. doi:10.1080/15384101.2016.1152433

4. Le Maitre CL, Freemont AJ, Hoyland JA. Accelerated cellular senescence in degenerate intervertebral discs: a possible role in the pathogenesis of intervertebral disc degeneration. *Arthritis Res Ther*. 2007;9(3):R45. doi:10.1186/ar2198

5. Childs BG, Gluscevic M, Baker DJ, et al. Senescent cells: an emerging target for diseases of ageing. *Nat Rev Drug Discov*. Oct 2017;16(10):718-735. doi:10.1038/nrd.2017.116

6. Elder SS, Emmerson E. Senescent cells and macrophages: key players for regeneration? *Open Biol*. Dec 2020;10(12):200309. doi:10.1098/rsob.200309

7. Sakamuri S, Sperling JA, Sure VN, et al. Measurement of respiratory function in isolated cardiac mitochondria using Seahorse XFe24 Analyzer: applications for aging research. *Geroscience*. Jun 2018;40(3):347-356. doi:10.1007/s11357-018-0021-3

8. Kurupati RK, Haut LH, Schmader KE, Ertl HC. Age-related changes in B cell metabolism. *Aging (Albany NY)*. Jul 8 2019;11(13):4367-4381. doi:10.18632/aging.102058

9. Pence BD, Yarbro JR. Aging impairs mitochondrial respiratory capacity in classical monocytes. *Exp Gerontol*. Jul 15 2018;108:112-117. doi:10.1016/j.exger.2018.04.008

10. Bahar ME, Hwang JS, Ahmed M, et al. Targeting Autophagy for Developing New Therapeutic Strategy in Intervertebral Disc Degeneration. *Antioxidants (Basel)*. Aug 14 2022;11(8)doi:10.3390/antiox11081571

11. Kang L, Xiang Q, Zhan S, et al. Restoration of Autophagic Flux Rescues Oxidative Damage and Mitochondrial Dysfunction to Protect against Intervertebral Disc Degeneration. *Oxid Med Cell Longev*. 2019;2019:7810320. doi:10.1155/2019/7810320

12. Zheng Z, Wang ZG, Chen Y, et al. Spermidine promotes nucleus pulposus autophagy as a protective mechanism against apoptosis and ameliorates disc degeneration. *J Cell Mol Med*. Jun 2018;22(6):3086-3096. doi:10.1111/jcmm.13586

13. Gong CY, Zhang HH. Autophagy as a potential therapeutic target in intervertebral disc degeneration. *Life Sci*. May 15 2021;273:119266. doi:10.1016/j.lfs.2021.119266

14. Bi F, Liu W, Wu Z, Ji C, Chang C. Antiaging Factor Klotho Retards the Progress of Intervertebral Disc Degeneration through the Toll-Like Receptor 4-NF-kappaB Pathway. *Int J Cell Biol*. 2020;2020:8319516. doi:10.1155/2020/8319516

15. Yi YY, Chen H, Zhang SB, Xu HW, Fang XY, Wang SJ. Exogenous Klotho ameliorates extracellular matrix degradation and angiogenesis in intervertebral disc degeneration via inhibition of the Rac1/PAK1/MMP-2 signaling axis. *Mech Ageing Dev*. Oct 2022;207:111715. doi:10.1016/j.mad.2022.111715

16. Zhang SJ, Yang W, Wang C, et al. Autophagy: A double-edged sword in intervertebral disk degeneration. *Clin Chim Acta*. Jun 1 2016;457:27-35. doi:10.1016/j.cca.2016.03.016

17. Gruber HE, Hoelscher GL, Ingram JA, Bethea S, Hanley EN, Jr. Autophagy in the Degenerating Human Intervertebral Disc: In Vivo Molecular and Morphological Evidence, and Induction of Autophagy in Cultured Annulus Cells Exposed to Proinflammatory Cytokines-Implications for Disc Degeneration. *Spine (Phila Pa 1976)*. Jun 1 2015;40(11):773-82. doi:10.1097/BRS.0000000000000865

18. Ni B, Shen H, Wang W, Lu H, Jiang L. TGF-beta1 reduces the oxidative stress-induced autophagy and apoptosis in rat annulus fibrosus cells through the ERK signaling pathway. *J Orthop Surg Res*. Jul 29 2019;14(1):241. doi:10.1186/s13018-019-1260-4

19. Ni BB, Li B, Yang YH, et al. The effect of transforming growth factor beta1 on the crosstalk between autophagy and apoptosis in the annulus fibrosus cells under serum deprivation. *Cytokine*. Dec 2014;70(2):87-96. doi:10.1016/j.cyto.2014.07.249

20. Ye W, Zhu W, Xu K, et al. Increased macroautophagy in the pathological process of intervertebral disc degeneration in rats. *Connect Tissue Res*. 2013;54(1):22-8. doi:10.3109/03008207.2012.715702

21. Ye W, Xu K, Huang D, et al. Age-related increases of macroautophagy and chaperone-mediated autophagy in rat nucleus pulposus. *Connect Tissue Res*. 2011;52(6):472-8. doi:10.3109/03008207.2011.564336

22. Chen J, Lin Z, Deng K, Shao B, Yang D. Tension induces intervertebral disc degeneration via endoplasmic reticulum stress-mediated autophagy. *Biosci Rep*. Aug 30 2019;39(8)doi:10.1042/BSR20190578

23. Zhao DW, Zhang J, Chen C, et al. Rejuvenation Modulation of Nucleus Pulposus Progenitor Cells Reverses Senescence-Associated Intervertebral Disc Degeneration. *Adv Mater*. Feb 2025;37(7)doi:10.1002/adma.202409979
